# Injectable click-crosslinked hydrogel containing resveratrol to improve the therapeutic effect in triple negative breast cancer^☆^

**DOI:** 10.1016/j.mtbio.2022.100386

**Published:** 2022-08-05

**Authors:** Gi Ru Shin, Hee Eun Kim, Hyeon Jin Ju, Jae Ho Kim, Sangdun Choi, Hak Soo Choi, Moon Suk Kim

**Affiliations:** a Department of Molecular Science and Technology, Ajou University, Suwon, 16499, South Korea; b Gordon Center for Medical Imaging, Department of Radiology, Massachusetts General Hospital and Harvard Medical School, Boston, MA, 02114, USA; c Research Institute, Medipolymers, Suwon, 16522, South Korea

**Keywords:** Triple-negative breast cancer, Resveratrol, Injectable hydrogel, Click-crosslinking, Intratumoral injection

## Abstract

Triple-negative breast cancer (TNBC) patients are considered intractable, as this disease has few effective treatments and a very poor prognosis even in its early stages. Here, intratumoral therapy with resveratrol (Res), which has anticancer and metastasis inhibitory effects, was proposed for the effective treatment of TNBC. An injectable Res-loaded click-crosslinked hyaluronic acid (Res-Cx-HA) hydrogel was designed and intratumorally injected to generate a Res-Cx-HA depot inside the tumor. The Res-Cx-HA formulation exhibited good injectability into the tumor tissue, quick depot formation inside the tumor, and the depot remained inside the injected tumor for extended periods. *In vivo* formed Res-Cx-HA depots sustained Res inside the tumor for extended periods. More importantly, the bioavailability and therapeutic efficacy of Res remained almost exclusively within the tumor and not in other organs. Intratumoral injection of Res-Cx-HA in animal models resulted in significant negative tumor growth rates (i.e., the tumor volume decreased over time) coupled with large apoptotic cells and limited angiogenesis in tumors. Therefore, Res-Cx-HA intratumoral injection is a promising way to treat TNBC patients with high efficacy and minimal adverse effects.

## Highlights

• Intratumoral injection was developed for treatment of triple negative breast cancer.
• Injectable formulation exhibited good injectability, quick depot formation.
• The formed depot remained inside the injected tumor for extended periods.
• Bioavailability and therapeutic efficacy of Res inside tumor were improved.
• *In vivo* formed depots resulted in significant negative cancer growth.

## Graphical abstract

**Figure:**
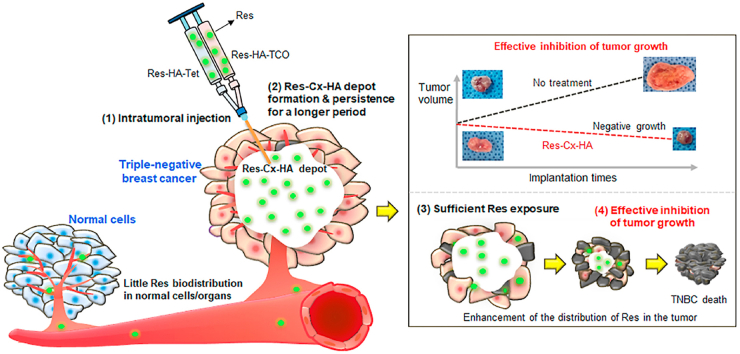

## 1 Introduction

Breast cancer cases are rapidly increasing due to its high metastasis and high systemic recurrence rate [1], which highlights the urgent need for new and more effective breast cancer treatments. Triple-negative breast cancer (TNBC) is defined as breast cancer in which the genes for estrogen receptor (ER), progesterone receptor (PR), and HER2/neu are not expressed [2]. TNBC patients account for approximately 20% of all breast cancer patients, and the majority of TNBC patients are young women or women with a mutation in the BRCA1 gene [3].

TNBC is characterized by high indices of mitosis and proliferation, HER2 overexpression, and poorly differentiated tumor features. Generally, even in its early stages, TNBC metastasizes to the brain and lungs, resulting in a very low survival rates [4]. Therefore, a new strategy for the treatment of TNBC is urgently needed.

Current TNBC treatments consist of administering general cancer drugs through systemic chemotherapy [5]. Furthermore, several classes of drugs such as VEGF inhibitors, tyrosine kinase inhibitors, and PARP inhibitors have been recently used as adjuvants for TNBC treatment, but have performed poorly in clinical trials [6]. Therefore, TNBC patients are generally very susceptible to chemotherapy. In some cases, however, there is no correlation between initial chemotherapy and overall survival, which makes it particularly complicated to select optimal chemotherapies.

Resveratrol (Res) is a Stilbene molecule (3,5,4′-trihydroxystilbene) belonging to the polyphenol family that is extracted from many natural plants, particularly grapes. Systemic oral administration of Res can result in its accumulation in various tissues (e.g., the liver, intestine, and stomach) and cancerous tumors through its circulation in the plasma [7].

Res induces apoptosis and thus not only possesses anticancer effects but also anti-metastasis effects, thereby reducing the side effects of chemotherapy [8]. Recent studies have also demonstrated that Res can attenuate chemotherapy-induced cardiotoxicity through various pathways.

Res has previously been used to treat TNBC and other types of cancer through systemic administration, including oral uptake, as well as intravenous and intradermal injection [9]. These studies demonstrated that Res possesses anticancer effects and low toxicity, in addition to reducing the side effects of chemotherapy and exerting antioxidant effects even in hypoxic conditions.

Nevertheless, the role of Res in the prevention and treatment of TNBC remains controversial, as Res has low bioavailability in human tissues due to its extremely short half-life (8–14 ​min) in plasma, although its metabolites circulate in the plasma for approximately 9.2 ​h [10]. Therefore, systemic Res administration (oral uptake, intravenous injection, and intradermal injection) leads to its rapid metabolization, resulting in low Res plasma concentrations. For this reason, multiple Res injections are often needed to maintain effective Res concentrations for tumor treatment.

Compared to conventional anticancer drug administration routes, intratumoral injection can achieve a high local concentration of the anticancer drug at the target tumor, including TNBC tumors [[11], [12], [13], [14], [15]]. In turn, this could improve drug efficacy and reduce or eliminate adverse effects. Therefore, recent studies have highlighted the importance of intratumoral delivery in antitumor therapy.

The microenvironment of the tumor matrix is among the most important factors to consider for the development of effective intratumoral injection therapies [[16], [17], [18]]. The microenvironment of the tumor matrix is characterized by an unorganized vascular network, a lack of functional lymphatic vessels, multiple extracellular matrix deposition, and abnormally high interstitial pressure. The microenvironment of the tumor matrix itself is a barrier that limits the diffusion and convection of the injected anticancer drug. Furthermore, the most aggressive cancer stem cells are often located at the core of the tumor. These cancer stem cells induce sustained cancer growth by regulating development, resistance, cancer progression, and metastasis [19]. Therefore, the removal of cancer stem cells in tumors via intratumoral injection is currently among the most important goals in cancer research. Intratumoral injection would thus provide an effective means to deliver anticancer agents directly to the tumor core.

Injectable *in situ*–forming hydrogels have been widely utilized in the biomedical field as drug carriers [[20], [21], [22], [23], [24]], as they allow for the easy and accurate incorporation of various biological factors by simply mixing them at room temperature, after which they can be injected in liquid form and easily form a hydrogel *in situ*.

There are several reports using natural materials such as biocompatible hyaluronic acid (HA), cellulose, silk, and collagen as injectable *in situ*-forming hydrogel drug carriers [[25], [26], [27], [28], [29], [30]]. Among them, an injectable HA hydrogel was reported to temporarily maintain its elastic and viscose integrity as a depot under physiological conditions [31]. This HA hydrogel exhibited a good capacity for water absorption and a high swelling ability upon water absorption. Therefore, an injectable HA hydrogel can create a three-dimensional depot that would enable the diffusion and convection of the injected anticancer Res inside the microenvironment of the tumor matrix. Furthermore, the injected HA depot itself could diminish the pore size of the tumor matrix and thus contribute to decreasing blood vessel formation within the tumor matrix [32,33].

Nevertheless, once injected, the HA hydrogel gradually and completely degrades under physiological conditions due to its short residence time [34]. A recent study demonstrated that the bioorthogonal Diels–Alder click reaction between tetrazine (Tet) and *trans*-cyclooctene (TCO) readily occurs even under physiological conditions and without an external catalyst [35]. To our knowledge, there are hardly any published studies on the formation of injectable click-crosslinked HA hydrogels as anticancer Res carriers [36]. Therefore, our study sought to evaluate the ability of Res-loaded Tet-modified HA (Res-HA-Tet) and Res-loaded TCO-modified HA (Res-HA-TCO) to form *in situ* click-crosslinked Res-loaded HA (Res-Cx-HA) hydrogels with long residence times within tumors. The Res-loaded HA-Tet and Res-loaded HA-TCO formulations can be easily injected using a syringe needle, thus allowing for the intratumoral administration of Res via injection. Therefore, we hypothesized that the Res-Cx-HA depot formed after intratumoral injection would effectively inhibit tumor growth.

**Fig. 1:**
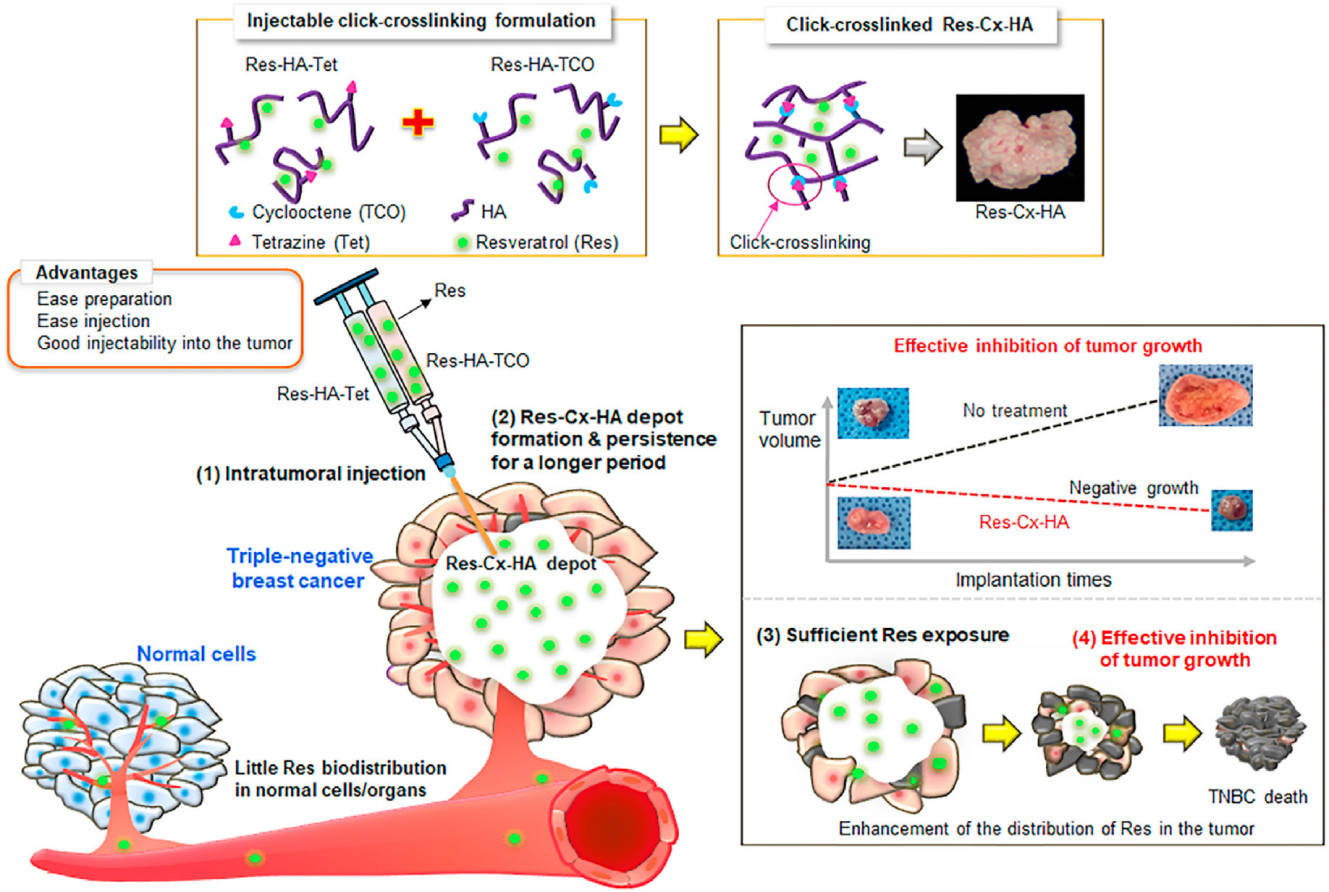
Schematic image of injectable, click-crosslinkable Res-HA-Tet and Res-HA-TCO formulations for intratumoral injection of Res and effective inhibition of tumor growth.

To the best of our knowledge, very few studies have conducted intratumoral injection of Res-HA-Tet or Res-HA-TCO formulations in test animals to assess their potential as TNBC treatments. Therefore, our study developed an efficient *in vivo* delivery strategy for the creation of intratumoral Res depots, which may have important implications for TNBC treatment (Fig. 1).

## 2 Experimental

### 2.1 Materials

Resveratrol (228.25 ​g/mol), 4-(4,6-dimethoxy-1,3,5-triazin-2-yl)-4-methyl-morpholinium chloride (DMTMM, Tokyo chemical industry, Tokyo, Japan), polysorbate 20 (Sigma, MO, USA), IR-783 (Sigma, MO, USA), methyltetrazine-PEG4-amine hydrochloride salt (Tet, Click Chemistry Tools, AZ, USA) and *trans*-cyclooctene-amine hydrochloride salt (TCO, Click Chemistry Tools, AZ, USA) were used in our experiments without further modification. Hyaluronic acid (HA) (1 ​MDa) was purchased from Kikkoman (Tokyo, Japan). The MDA-MB-231 breast cancer cell line was purchased from the Korea Cell Line Bank (Seoul, Korea). Rosewell Park Memorial Institute medium (RPMI 1640), 0.05% trypsin-EDTA, Dulbecco's phosphate-buffered saline (DPBS), penicillin-streptomycin (PS), and fetal bovine serum (FBS) were purchased from Gibco BRL (Carlsbad, CA, USA).

### 2.2 Preparation of HA-TCO and HA-Tet

HA solutions were prepared by dissolving HA powder (100 ​mg) in 10 ​mL deionized water (DW). DMTMM (70 ​mg, 0.26 ​mmol) was added to the HA solution and then stirred for 30 ​min to activate the carboxyl group of HA. TCO (20 ​mg, 0.076 ​mmol) and Tet (27 ​mg, 0.074 ​mmol) were individually added to the HA solution activated by DMTMM and then allowed to react for 24 ​h. The reaction solution was dialyzed for 72 ​h to remove the unreacted TCO or Tet, and then were lyophilized in a freeze dryer (FD 8508, Ilshinlab, Daejeon, Korea). The yields of HA-Tet and HA-TCO were 95.9% and 86.2%, respectively. The structures of the obtained compounds were confirmed by ^1^H NMR spectroscopy. The introduction of Tet and TCO was confirmed via elemental analysis of the amine groups in HA-Tet (C: 42.6%, H: 6.1%, N: 6.2%) and HA-TCO (C: 42.9%, H: 6.0%, N: 4.95%).

### 2.3 Preparation of NIR-HA, NIR-HA-Tet, and NIR-HA-TCO

IR-783 infrared dye (250 ​mg, 0.33 ​mmol) was added to a solution of sodium azide (30 ​mg, 0.5 ​mmol) in dimethylformamide (10 ​mL), and the mixture was stirred at 65 ​°C for 24 ​h. Propargyl amine (42.6 ​mg, 0.66 ​mmol), copper sulfate (110 ​mg, 0.66 ​mmol), and ascorbic acid (240 ​mg, 1.32 ​mmol) were added to the reacted IR-783-N3 solution, after which the mixture was stirred for 24 ​h at 25 ​°C. Ether was added to the reaction mixture to precipitate IR-783-NH_2_. The IR-783-NH_2_ was collected via filtration and dried under vacuum. The COOH groups in pure HA, HA-Tet, and HA-TCO in an aqueous 10 ​mg/mL solution were activated in the presence of DMTMM (16 ​mg, 0.057 ​mmol) for 1 ​h at 25 ​°C. IR-783-NH_2_ (30 ​mg, 0.038 ​mmol) was added to the activated HA-Tet and HA-TCO solutions, and the mixtures were stirred for 24 ​h. Unreacted IR-783-NH_2_ was removed via dialysis for three days using membranes with a molecular weight cutoff (MWCO) of 3.5–5 ​kDa to obtain pure HA-NIR, HA-NIR-Tet, and HA-NIR-TCO.

### 2.4 Preparation of Res-HA and Res-Cx-HA formulations

The ratio of Res to HA or Cx-HA was 5 ​mg/mL to 40 ​mg/mL. For the preparation of Res-HA, Res (5 ​mg) was added to 40 ​mg of HA in 1 ​mL of PBST [4% Tween in phosphate-buffered saline (PBS)] and gently vortexed. The Res-HA was subsequently loaded into a single-barrel syringe. To prepare Res-Cx-HA, 2.5 ​mg of Res was added to 20 ​mg of HA-Tet in 0.5 ​mL of PBST and 20 ​mg of HA-TCO in 0.5 ​mL of PBST. Res-loaded HA-Tet (Res-HA-Tet) and Res-loaded HA-TCO (Res-HA-TCO) were loaded separately into the compartments of a dual-barrel syringe.

### 2.5 Rheological characterization of HA and Cx-HA (from HA-Tet and HA-TCO)

HA, HA-Tet, and HA-TCO solutions were individually prepared at a 20 ​mg/mL concentration. The HA solution was loaded into a single-barrel syringe, whereas the HA-Tet and HA-TCO solutions were separately loaded into the compartments of a dual-barrel syringe. The rheological properties of the formulations were analyzed using an MCR 102 rheometer (Anton Paar, Austria), which was equipped with a temperature-controlled bottom plate and a parallel 25.0 ​mm stainless steel plate. All measurements were performed at 25 ​°C with a 0.3 ​mm gap. The oscillation frequency sweep was measured at 1 ​Hz with a fixed 2% strain. The rheological properties were analyzed using Rheoplus/32 version V3.21 (Anton Paar).

### 2.6 Measurement of injection force of the injectable HA and Cx-HA formulations (from HA-Tet and HA-TCO without and with res)

HA only, HA-Tet, and HA-TCO or Res-HA-Tet and Res-HA-TCO solutions were loaded separately into the compartments of a dual-barrel syringe. Injection force was determined using a universal testing machine (H5KT, Tinius-Olsen, Horsham, PA, USA) at a 60 ​mm/min rate up to a maximum load of 500 ​N against the syringe (needle gauge: 23 ​G; 0.337 ​mm inner diameter, 25.4 ​mm length). The injection force of each solution was measured in compression mode using the Horizon software (Tinius Olsen, Rock Hill, SC, USA). The maximum force of each formulation was determined from force-displacement plots.

### 2.7 *In vitro* antiproliferative efficacy of the injectable formulations

MDA-MB-231 ​cells (10^5^ per well) were cultured in the bottom chambers of 24-well Transwell plates (SPL Life Science, 0.4 ​μm pore size, Pocheon-si, Korea) and incubated for 1 day at 37 ​°C in a humidified incubator containing 5% CO_2_. Res-HA and Res-Cx-HA formulations (0.15 ​mg Res concentration per well) were added to the upper chambers of 24-well Transwell plates. The transwell medium was replaced with fresh culture media on day 1.

On days 1, 2, and 3, MDA-MB-231 tumor cells were stained by Hoechst 33342 (Invitrogen, MA, USA). Fluorescent images of MDA-MB-231 tumor cells were obtained at control (no treatment), Res, Res-HA, and Res-Cx-HA. Additionally, on days 1, 2, and 3, the viability of the MDA-MB-231 ​cells was evaluated using 3-(4,5-dimethylthiazol-2-yl)-2,5-diphenyltetrazolium bromide (MTT; Sigma- Aldrich Co., St Louis, MO, USA). All experiments were conducted in triplicate. Briefly, 100 ​μL of MTT solution (50 ​μg/mL in DPBS) was added to each well plate, and the plates were incubated at 37 ​°C for 4 ​h and 30 ​min. The resulting violet formazan precipitate was solubilized by adding 500 ​μL DMSO and shaking for 20 ​min. The solutions were placed in 96-well plates and read using a microplate reader (E-max, Molecular Devices, Sunnyvale, CA, USA). The optical density of each well was measured at a 570 ​nm wavelength. All experiments were performed at least three times and the results are reported as mean ​± ​standard deviation (SD).

### 2.8 Animal experiments

The protocol for the animal experiments was approved by the Institutional Animal Experiment Committee (Approval no. 2020–0041) of the Ajou University School of Medicine. All experiments were performed using six-week-old female nude mice (20–22 ​g) in accordance with the guidelines for the care and use of animals for experimental and scientific purposes.

### 2.9 *In vivo* imaging of injectable formulations

NIR-HA was loaded into single-barrel syringes with 23-gauge needles. NIR-HA-Tet and NIR-HA-TCO were loaded into separate compartments of a dual-barrel syringe with a 23-gauge needle. Next, 50 ​μL of each formulation was injected subcutaneously into the dorsa of nude mice or was injected directly into the tumor after anesthetizing the mice with 1:1 Zoletil-Rompun solution (60 ​μL/kg). The formulations contained the same concentration of NIR dye (20 ​mg/mL). NIR fluorescence images of the HA hydrogels were obtained at a 730 ​nm excitation wavelength. The emitted light was filtered through a 750–825 ​nm bandpass filter with an exposure time of 1500 ​ms and a gain of 1 using a dichroic MgF_2_ fused silica cube filter. Images were captured at specified time points using a FOBI fluorescence in-vivo imaging system (NeoScience, Suwon, Korea).

### 2.10 *In vivo* anti-tumor activity

The tumor model was established by subcutaneous inoculation of MDA-MB-231 ​cells (1 ​× ​10^7^ ​cells, in a 0.1 ​mL suspension) into the abdomen of each female nude mice after anesthetizing them with 1:1 Zoletil-Rompun (60 ​μL/kg). The time when the solid tumor volume reached 79 ​± ​12 ​mm^3^ was defined as day 0. The animals were assigned to four experimental groups: 1) no treatment group, 2) Res, 3) Res-HA, and 4) Res-Cx-HA (2.5 ​mg Res concentration per formulation). On day 0, 50 ​μL of Res and Res-HA was injected directly into the tumor using a 23-gauge needle and a single-barrel syringe. Res-HA-Tet and Res-HA-TCO solutions were loaded separately into the compartments of a dual-barrel syringe.

Antitumor activity was assessed by measuring tumor diameters in two dimensions with Vernier calipers at predefined days. The tumor volume (*V*) was calculated according to the following formula: *V* ​= ​0.52 ​× ​width ​× ​depth ​× ​height.

For pharmacokinetics, experimental animals were sacrificed on days 1, 6, 12, and 18 via cervical dislocation, and the organs (small intestine, colon, stomach, lungs, kidney, spleen, liver, and heart) were harvested immediately. Each organ was immediately frozen at −70 ​°C for 24 ​h, then freeze-dried for 7 ​d. The dehydrated tumors and organs were homogenized in 0.1 ​N HCl using a T10 basic ULTRA-TURRAX Homogenizer (IKA, Werke GMBH, Germany) at 35,000 ​rpm. Each sample was mixed with an equal volume of 40% (w/v) ZnSO_4_ and mobile phase (as described in the previous section), then incubated again at 37 ​°C for 15 ​min. The amount of Res in the supernatant, which was obtained after centrifugation at 2000 ​rpm for 10 ​min. The amount of Res in tumor and each organ was determined using high-performance liquid chromatography (HPLC; Agilent 1200 series, Waldbronn, Germany). The HPLC analysis conditions used for the measurement were measured at a flow rate of 1 ​mL/min at a maximum absorption wavelength of 306 ​nm using a column (Osaka soda, capcell pak C18 UG120 S-5) at 40 ​°C using 30% ACN as a mobile phase. Three independent experiments were performed for each organ. The Res amount was calculated by comparing it with a standard calibration curve prepared with Res prepared at a known concentration.

### 2.11 Histological analysis

The experimental mice were euthanized on days 1, 12, and 18 after the initial injection, and the tumors were excised from the subcutaneous abdomen. The tissues were immediately fixed with 10% formalin and embedded in paraffin. The embedded specimens were then sectioned (4 ​μm) along the longitudinal axis of the tumor and incubated at 70 ​°C for 12 ​h to remove excess paraffin. The slides were further deparaffinized with xylene twice and sequentially hydrated using 100%, 95%, 80%, and 70% ethyl alcohol. For hematoxylin and eosin (H&E) staining, the samples were washed in running tap water and stained with H&E. Afterward, the stained slides were mounted with mounting medium (Fisher Chemical, Geel, Belgium).

Apoptotic cells were identified using a terminal deoxynucleotidyl transferase dUTP nick-end labeling (TUNEL) assay kit (In Situ Cell Death Detection Kit, Roche, Germany) following the manufacturer's instructions. Briefly, the slides were deparaffinized at 70 ​°C, hydrated, and washed three times with PBS-T (0.1% Tween®20 in PBS) for 10 ​min per wash.

Afterward, the tissues were placed in citrate buffer and microwave-treated for 1 ​min. Next, the mixture was incubated at 37 ​°C for 60 ​min with a mixture of label solution and enzyme solution (9:1). For nuclear staining, the slides were counterstained with 4′,6-diamidino-2-phenylindole (DAPI), then mounted with a mounting solution (Vectashield, Burlingame, CA, USA).

The slides were then hydrated for 5 ​min for cleaved caspase-3 (CCP-3) (Cell signaling technology, Danvers, MA, USA) staining. After treatment with citrate buffer solution at 120–130 ​°C for 10 ​min, the slides were washed with PBS and PBS-T (0.05% Tween 20 in PBS), then blocked in 5% bovine serum albumin and 5% horse serum (HS) in PBS at 37 ​°C for 90 ​min. Next, the samples were treated with cleaved caspase-3 primary antibodies (Cell signaling technology, Danvers, MA, USA) at a 1:2000 dilution for 16 ​h at 4 ​°C. After washing the slides with PBS and PBS-T, secondary anti-body treatment (mouse anti-mouse Alexa Fluor 488, Eugene, OR) was performed at room temperature for 3 ​h in the dark, after which the slides were washed with PBS and PBS-T for 20 ​min. The slides were then treated with diluent solution (Dako, Carpinteria, USA) (1:200) for nuclear staining, after which they were counterstained with 4′,6-diamidino-2-phenylindole (DAPI). The stained slides were then mounted in a mounting medium (Vectashield, Burlingame, CA, USA).

For CD31 staining, the slides were hydrated for 5 ​min, treated with citrate buffer solution at 120–130 ​°C for 10 ​min, washed with PBS and PBS-T (0.05% Tween 20 in PBS), and then blocked for 90 ​min with 5% bovine serum albumin (BSA; Roche, Penzberg, Germany) and 5% horse serum (HS; Gibco, Auckland, New Zealand) in PBS at 37 ​°C. Afterward, the samples were treated at 4 ​°C for 16 ​h with CD31 primary antibodies (Abcam, Cambridge, UK) diluted in diluent solution (DAKO; Glostrup, Denmark) at a 1:200 ratio. After washing the slides with PBS and PBS-T, secondary anti-body treatment (mouse anti-mouse Alexa Fluor 488, Eugene, OR) was performed at room temperature for 3 ​h in the dark, after which the slides were washed with PBS and PBS-T for 20 ​min. After being counterstained with a green dye in diluent solution (1:200) for nuclear staining, the slides were counterstained with 4′,6-diamidino-2-phenylindole (DAPI, Roche, Mannheim, Germany), after which the stained slides were counterstained with mounting medium (Vectashield, Burlingame, CA, USA). All staining and fluorescence images were obtained using a slide scanner (ZEISS Axio Scan. Z1, Carl Zeiss Microscopy GmbH, Jena, Germany) and analyzed with the ZEN 2009 software (Carl Zeiss Microscopy GmbH, Jena, Germany). The stained images were quantitatively analyzed using the ImageJ program. The quantification for positive cells of CD31, TUNEL and CCP-3, and total DAPI numbers was determined on each image using the ImageJ software (National Institutes of Health, Bethesda, MD, USA) and individually calculated as follows: Positive ratio (%) ​= ​Each positive cells/total DAPI numbers.

### 2.12 Statistical analyses

Tumor size was assessed in experimental groups with *n* ​= ​3 for all data points, and Res content was obtained from independent experiments in which all experimental groups were tested in triplicate. Staining analysis results were independently obtained in triplicate by different persons, and all data are presented as mean ​± ​SD. Data from each experiment were examined using one-way analysis of variance (ANOVA) with Bonferroni's multiple comparisons. All statistical analyses were performed using SPSS 12.0 (IBM Corporation, Armonk, NY, USA).

## 3 Results

### 3.1 Preparation and characterization of injectable Cx-HA hydrogel formulation

The injectable click-crosslinking HA hydrogels were prepared as described in a previous study [34,35]. Briefly, the carboxylic groups in HA were first activated with DMTMM and then allowed to react with Tet and TCO to obtain HA-Tet and HA-TCO, respectively (Figure S1). The characteristic peaks of HA-Tet and HA-TCO were seen in the ^1^H NMR spectra (Figure S2), and the formation of HA-Tet and HA-TCO was confirmed through elemental analysis as described previously. The degrees of Tet and TCO substitution on the carboxylic groups in HA were determined to be 95.9% for HA-Tet and 86.2% for HA-TCO based on their C/N ratios.

**Fig. 2:**
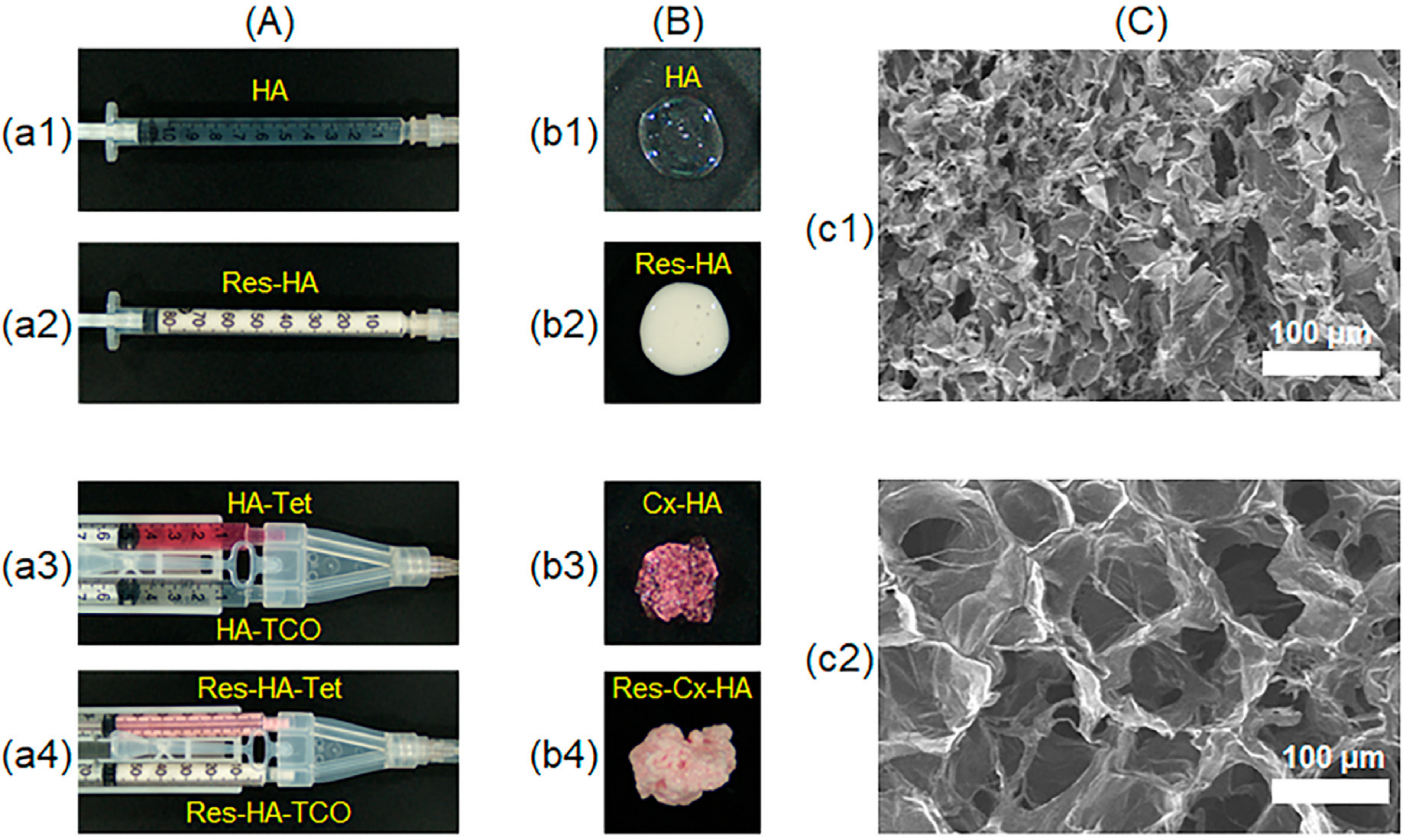
Images of (A) injectable formulations of (a1) HA and (a2) Res-HA in a single-barrel syringe, (a3) HA-Tet and HA-TCO and (a4) Res-HA-Tet and Res-HA-TCO in a dual-barrel syringe, (B) the formed depot of (b1) HA, (b2) Res-HA, (b3) Cx-HA, and (b4) Res-Cx-HA after injection through a 23-gauge needle and (C) SEM images of the formed (c1) HA and (c2) Cx-HA depot (scale bar: 100 ​μm).

HA alone or HA-Tet and HA-TCO with and without Res were easily solubilized in PBS. Each solution was loaded into either single-barrel or dual-barrel syringes (Fig. 2). Prior to injection, each formulation was stored in liquid form at room temperature in their respective single-barrel and dual-barrel syringes (Fig. 2A).

HA alone formed a viscose gel upon being pushed from the single-barrel syringe. In contrast, HA-Tet and HA-TCO with and without Res quickly click-crosslinked to form Cx-HA or Res-Cx-HA when pushed from the dual-barrel syringe (Fig. 2B). This indicated that the Cx-HA or Res-Cx-HA formed immediately upon mixing the HA-Tet and HA-TCO solutions with or without Res.

The HA and Cx-HA formed after injection was frozen in liquid nitrogen, freeze-dried, and observed by SEM (Fig. 2C). The HA appeared to form clusters in the SEM micrographs, whereas the cross-sectioned Cx-HA exhibited an interconnected structure with a pore diameter of 40–100 ​μm. This result indicated that Cx-HA could provide useful cavities for the penetration of biological medium into the hydrogel.

### 3.2 Rheological and injectable properties of injectable Cx-HA hydrogel formulations

**Fig. 3:**
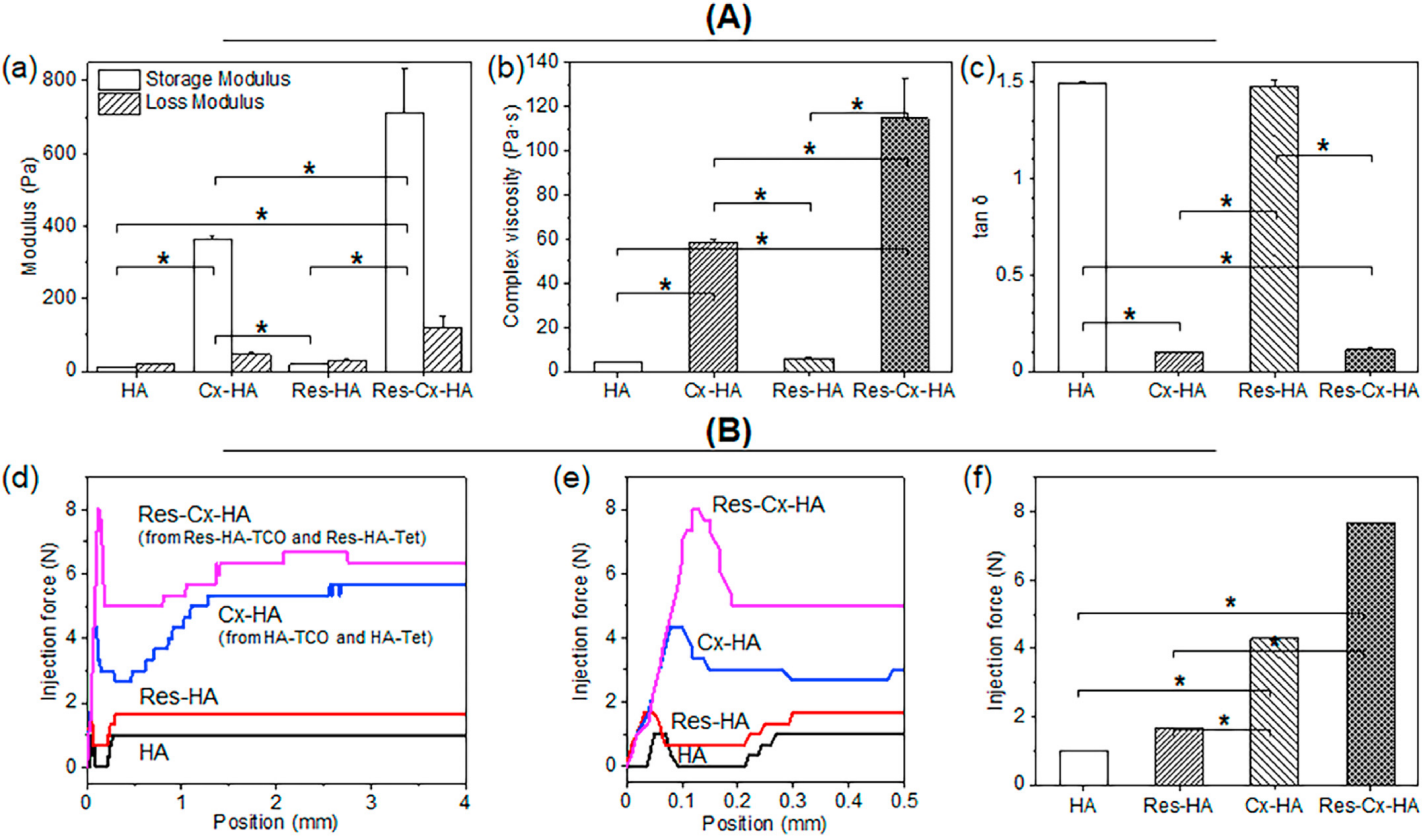
(**A**) Rheological characterization of HA and Cx-HA hydrogels with and without Res; (a) storage and loss modulus, (b) complex viscosity analysis, (c) tan ​δ value measurements (∗*p* ​< ​0.001). (**B**) Injectability of HA and Cx-HA hydrogels with and without Res; (d) force versus injection distance of HA and Res-HA in a single-barrel syringe, HA-Tet and HA-TCO with and without Res in a dual-barrel syringe, (e) force versus enlarged injection distance of each formulation, and (f) maximum force of each formulation (∗*p* ​< ​0.001).

The rheological properties of HA, Cx-HA, Res-HA, and Res-Cx-HA were evaluated within a 0.1–10 ​Hz frequency range (Fig. 3A). There was little difference between the storage (*G*′) and low loss (*G*″) moduli of HA and Res-HA. In contrast, the Cx-HA or Res-Cx-HA hydrogel generated by the injection of HA-Tet and HA-TCO or Res- HA-Tet and Res-HA-TCO exhibited high storage (G′) and low loss (G″) moduli.

The viscosity of Cx-HA and Res-Cx-HA was 15- or 20-fold higher than that of HA or Res-HA. Furthermore, Res-Cx-HA exhibited a 2-fold higher complex viscosity than that of Cx-HA. These results indicated that the click-crosslinking reaction increased viscosity and Res slightly affected the viscoelastic properties of Cx-HA. The phase angles (tan *δ*s) of Cx-HA and Res-Cx-HA calculated from *G*″/*G*′ were 0.09 and 0.11 (i.e., substantially less than 1). Taken together, our findings indicated that Cx-HA and Res-Cx-HA were significantly stiffer and more hydrogel-like than HA and Res-HA, although Res slightly modified the basic properties of Cx-HA.

Next, the extrusion of each formulation for HA and Res-HA in a single-barrel syringe and HA-Tet and HA-TCO with and without Res in a dual-barrel syringe was individually evaluated to assess the injectability of the solutions during the click-crosslinking process (Figure S3). Extrusion proceeded without clogging a 23-gauge needle until the solutions of all formulations were consumed. Particularly, HA-Tet and HA-TCO with and without Res were extruded without clogging for 30 ​s. Moreover, the extruded HA-Tet and HA-TCO with and without Res rapidly formed Cx-HA and Res-Cx-HA. This injection behavior indicated that the HA-Tet and HA-TCO formulation with and without Res can be suitable for intratumoral injection.

Finally, the injection force of HA and Res-HA in the single-barrel syringe and HA-Tet and HA-TCO with and without Res in a dual-barrel syringe were measured using a universal testing machine (Fig. 3B). All formulations showed similar force-distance curve behavior in three aspects: (1) the resistance maximum force to move the filled formulation by the syringe plunger (maximum peak shape); (2) the force that moves in proportion to the force applied to the syringe after restoring the original formulation viscosity from the maximum force (valley peak shape); (3) final force for equivalent movement of the formulation in the syringe (equilibrium horizontal shape).

The maximum force applied to a syringe plunger during the injection of a formulation via a needle was determined from the force-distance curve obtained with the Horizon software. The maximum injection forces of HA and Res-HA were 1 and 1.68 ​N, respectively, both of which were considered low. The injection force of Res-HA was ∼1.7-fold higher than that of HA without Res. Additionally, Res-Cx-HA showed a 1.8-fold higher injection force than that of Cx-HA. This behavior was highly consistent with the results from the rheological measurement.

### 3.3 Anti-proliferative effects of injectable formulations

**Fig. 4:**
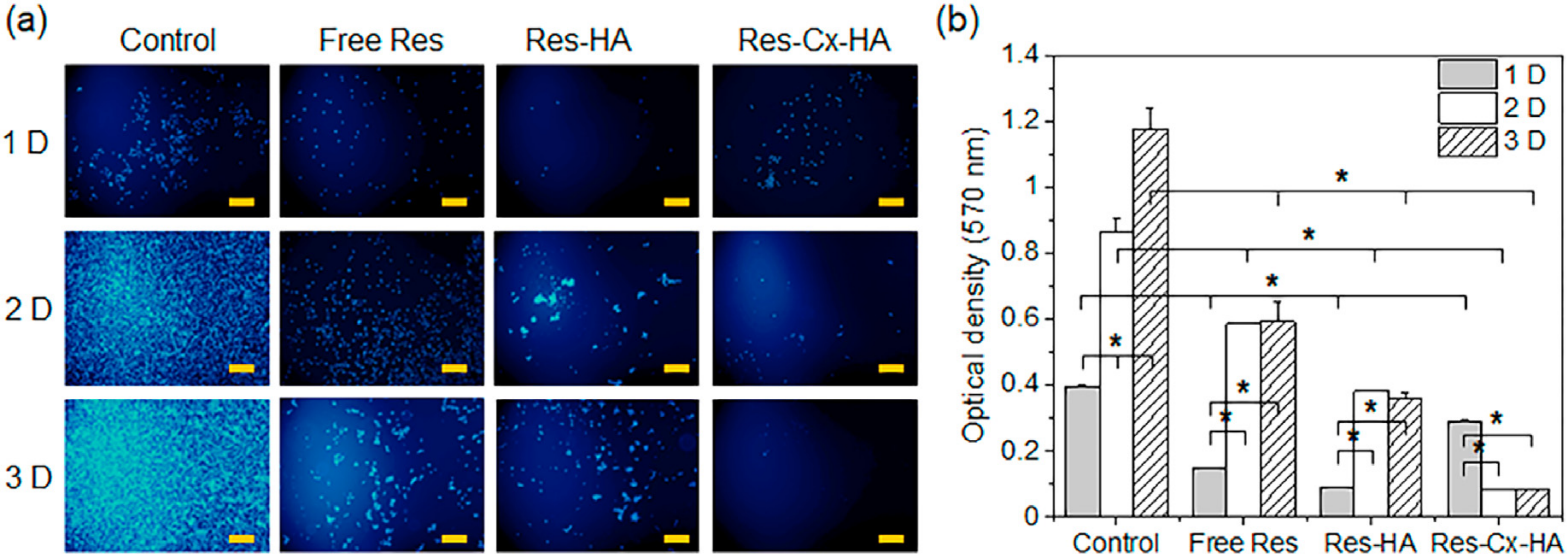
*In vitro* viability of MDA-MB-231 ​cell line in all formulations measured in this study. (a) Fluorescent images showing the morphology of MDA-MB-231 treated with all formulations after 1, 2, and 3 days (Scale bar: 200 ​μm) and (b) MTT assay results (∗*p* ​< ​0.005).

Fluorescent images of MDA-MB-231 tumor cells were obtained after exposure to control (no treatment), Res, Res-HA, and Res-Cx-HA (Fig. 4a). Blue fluorescence was indicative of Hoechst 33342-stained nuclei in the MDA-MB-231 ​cells.

The control (no treatment) cells exhibited a steady increase in blue fluorescence from days 1–3, indicating an increase in MDA-MB-231 ​cell populations. The cells treated with Res showed little blue fluorescence on day 1 but blue fluorescence increased on days 2 and 3. The cells treated with Res-HA exhibited low levels of blue fluorescence on day 1, which slightly increased thereafter. However, the blue fluorescence was lower than that of the control and Res groups. This result suggests that the Res released from Res-HA inhibited cell proliferation.

In the cells treated with Res-Cx-HA, the blue fluorescence decreased with culture time. These results suggest that the sustained release of Res from Res-Cx-HA almost entirely inhibited the proliferation of the MDA-MB-231 ​cells.

The anti-proliferative activities of the control (no treatment), Res, Res-HA, and Res-Cx-HA treatments were assessed via the MTT assay (Fig. 4b). The control cells exhibited a time-dependent increase in populations. Specifically, cell numbers increased by ∼220% on day 2 and ∼300% on day 3 compared to the cell numbers on day 1 (∗*p* ​< ​0.005).

The cell viability in the Res and Res-HA groups was <40% and 25% on day 1 compared to the control, indicating that Res had anti-proliferative effects on MDA-MB-231 tumor cells. However, the cells recovered and proliferated thereafter (∗*p* ​< ​0.005). Compared to the Res treatment, Res-HA only slightly inhibited cell proliferation on day 3.

The cell viability during exposure to Res-Cx-HA was approximately 70% on day 1 compared to the control on day 1. This cell viability rate was higher than that of the Res or Res-HA treatments because the Cx-HA slowed the release of Res. However, cell viability rapidly decreased to approximately 25% on days 2 and 3 compared to that of Res-Cx-HA on day 1. These results indicate that Res-Cx-HA can produce nearly complete anti-proliferative effects due to the sustained release of Res from its matrix.

### 3.4 *In vivo* persistence of HA, Cx-HA, Res, Res-HA, and Res-Cx-HA

**Fig. 5:**
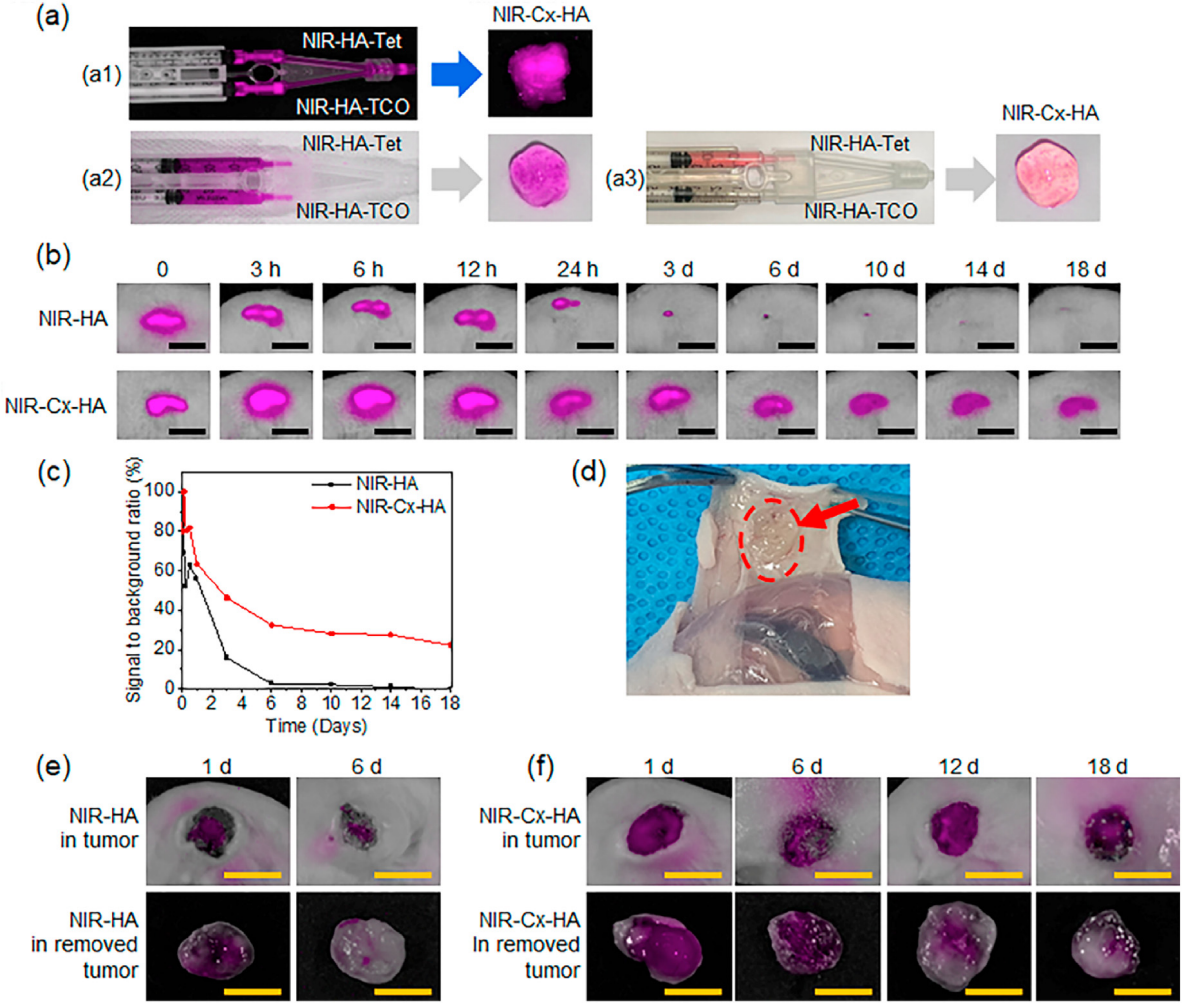
Near-infrared (NIR) observations. (a) NIR images [(a1) black background, (a2) white background, and (a3) brightness] of NIR-HA-Tet and NIR-HA-TCO loaded into a dual-barrel syringe and the formed NIR-Cx-HA. (b) NIR image after subcutaneous injection with NIR-HA or NIR-Cx-HA for 18 days (scale bar ​= ​1 ​cm). (c) Time after injection of NIR-HA or NIR-Cx-HA versus signal-to-background ratio (SBR) determined at each time point. (d) NIR-Cx-HA image of the formed NIR-Cx-HA (indicated with a circle) after 18 days. (e) NIR images of NIR-HA in tumors of animals and the removed tumor after 1 and 6 days. (f) NIR images of NIR-Cx-HA in tumors of animals and the removed tumor after 1, 6, 12, and 18 days (scale bar ​= ​1 ​cm).

NIR images of NIR fluorescence-labeled HA or Cx-HA were captured to assess the *in vivo* persistence of HA and Cx-HA hydrogels, as this is a critical factor that determines the formation of an intratumoral Res depot. The prepared NIR-HA-Tet and NIR-HA-TCO solutions were each loaded into the compartment of a dual-barrel syringe as illustrated in Fig. 5a. The NIR-HA-Tet and NIR-HA-TCO solutions formed NIR-Cx-HA when extruded from the syringe.

The prepared NIR-HA or NIR-HA-Tet and NIR-HA-TCO were individually subcutaneously injected into nude mice. Immediately after the injection, a NIR-HA or NIR-Cx-HA hydrogel formed at the injection site (Fig. 5b). NIR fluorescent images of NIR-HA gradually decreased and faint NIR images could be captured until day 10 but signals were no longer detected at 18 days. In contrast, NIR fluorescent images of the Cx-HA hydrogel could be successfully captured *in vivo* for at least 18 days (Fig. 5b,c,d). This result indicated that the Cx-HA hydrogel persisted *in vivo* for a much longer period compared to HA.

To investigate the persistence of NIR-HA or NIR-Cx-HA in the tumor region after *in vivo* intratumoral injections, formulations of NIR-HA or NIR-HA-Tet and NIR-HA-TCO were intratumorally injected. Fluorescence images were acquired from injected tumors and removed tumors after 1, 6, 12, and 18 days (Fig. 5e and f).

NIR-HA produced a pink NIR image one day after injection both in the injected tumor and the removed tumor. After 6 days, NIR fluorescence became faint or undetectable, suggesting fast clearance of NIR-HA from the intratumorally injected tumor. This indicated that NIR-HA had only a short residence time and the nearly complete and rapid degradation by hyaluronidase.

A pink NIR fluorescence signal was detected in intratumorally NIR-Cx-HA injected tumor sections after one day, indicating the formation of a NIR-Cx-HA depot in the intratumorally injected tumor site. Dispersed pink fluorescence was observed in the tumor after 6 and 12 days. Additionally, our findings confirmed that Cx-HA was accurately injected into the tumor. Pink NIR fluorescence was detected even on day 18, albeit at low intensities. This data indicated that Cx-HA could act as a depot for Res in the intratumorally injected tumor site, in addition to having a long residence time inside the tumor compared with HA. Collectively, our results confirmed that Cx-HA forms a suitable Res depot in the intratumorally injected tumor site for a predefined experimental period.

### 3.5 *In vivo* anti-tumor efficacy of Res, Res-HA, and Res-Cx-HA

Formulations of saline (control), Res, Res-HA, and Res-Cx-HA were prepared to conduct *in vivo* intratumoral injections. Each formulation was injected directly into the tumor of xenografted animals. No significant difference in weight was observed in the animals with *in vivo* intratumoral injections of control, Res, Res-HA, and Res-Cx-HA solutions (Figure S4).

**Fig. 6:**
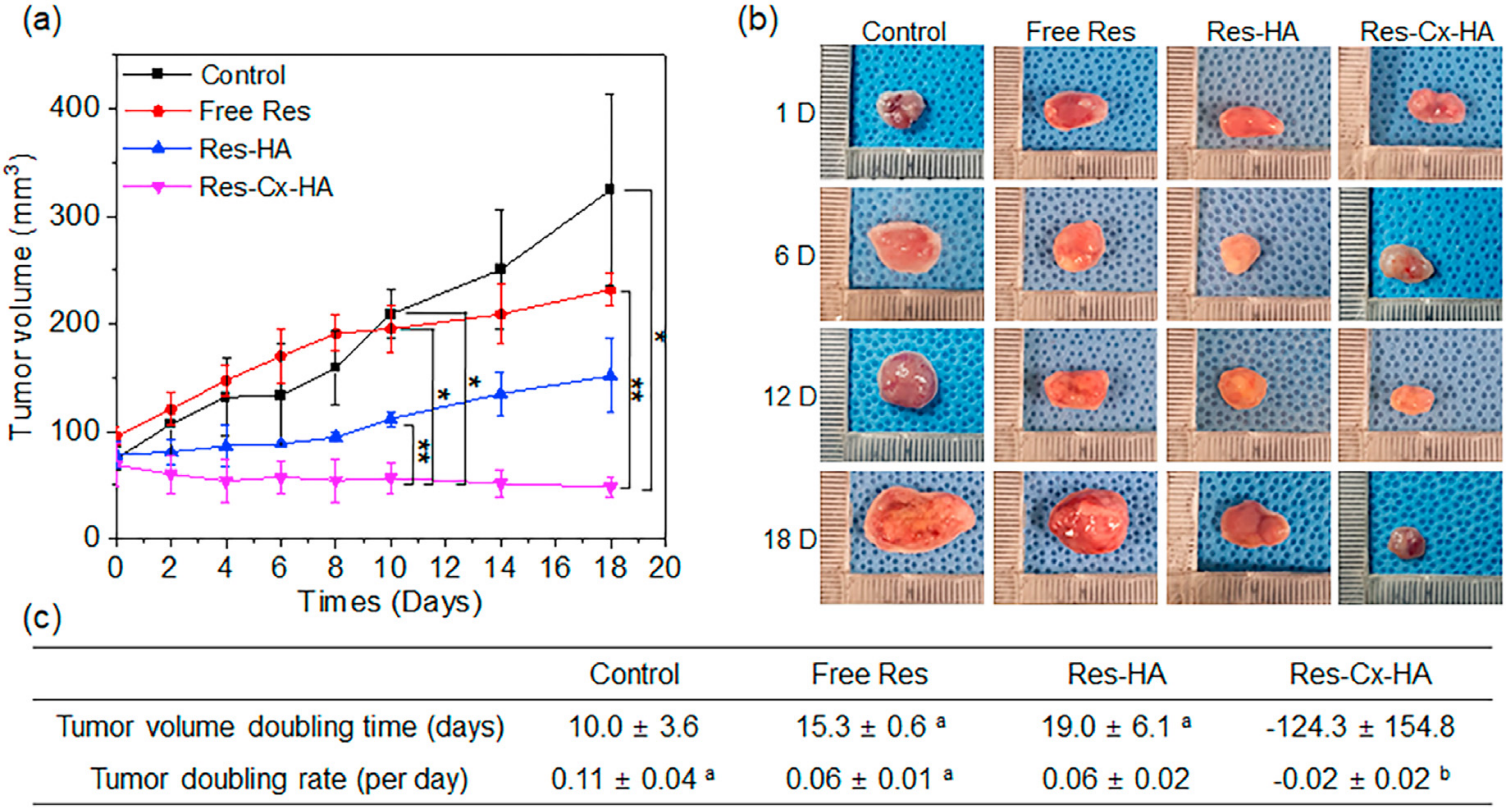
(a) Changes in tumor volume (∗*p* ​< ​0.005, ∗∗*p* ​< ​0.05, Free Res, Res-HA and Res-Cx-HA versus the control at 10 and 18 days), (b) images of the removed tumor (the images were taken from mice bearing MDA-MB-231 tumor cell xenografts) and (c) tumor volume doubling time and tumor volume growth rate after injection of control, Res, Res-HA, and Res-Cx-HA solutions (^a^*p* ​< ​0.1 and ^b^*p* ​< ​0.05 versus control).

The antiproliferative activities of the formulations were assessed by monitoring changes in the tumor volume (Fig. 6a and b). The average tumor size on day 0 was 79 ​± ​12 ​mm^3^. After 18 days, the size of the control tumors increased 4.2-fold, whereas the Res and Res-HA mice exhibited only 2.4- and 1.9-fold increases, respectively. These results indicate that free Res or Res-HA suppressed tumor growth, although the effect was relatively subtle. In contrast, Res-Cx-HA reduced the tumor volume by approximately 14 times compared to the original tumor size, which highlighted the strong antitumor activity of this treatment.

Tumor volume doubling times and tumor growth rates are summarized in Fig. 6c. The control showed a short doubling time of 10 days and a rapid tumor growth rate of 0.11 ​mm^3^ per day. Res and Res-HA extended the doubling time to 15 and 19 days, respectively, and decreased the tumor growth rate to 0.06 ​mm^3^ in both groups. Furthermore, Res and Res-HA exhibited a slight antitumor activity compared to the control; however, even this subtle activity appeared to decrease over time. Overall, Res and Res-HA were able to suppress tumor size more effectively than the control. Nevertheless, Res could not be retained for a long time due to its rapid release rates. This happened because Res rapidly escaped from the injected tumor.

In mice receiving intratumoral injection of Res-Cx-HA, the average tumor volume doubling time was −124 days, and the growth rate was −0.02 ​mm^3^ per day. These negative values indicated that the tumor volume decreased over time. Particularly, the Res-Cx-HA produced greater suppression of tumor growth than free Res or Res-HA. This was likely caused by the sustained Res release from the Res-Cx-HA. As expected, our results demonstrated that the formed Res-Cx-HA depot after intratumoral injection effectively inhibited tumor growth.

### 3.6 Biodistribution of Res

**Fig. 7:**
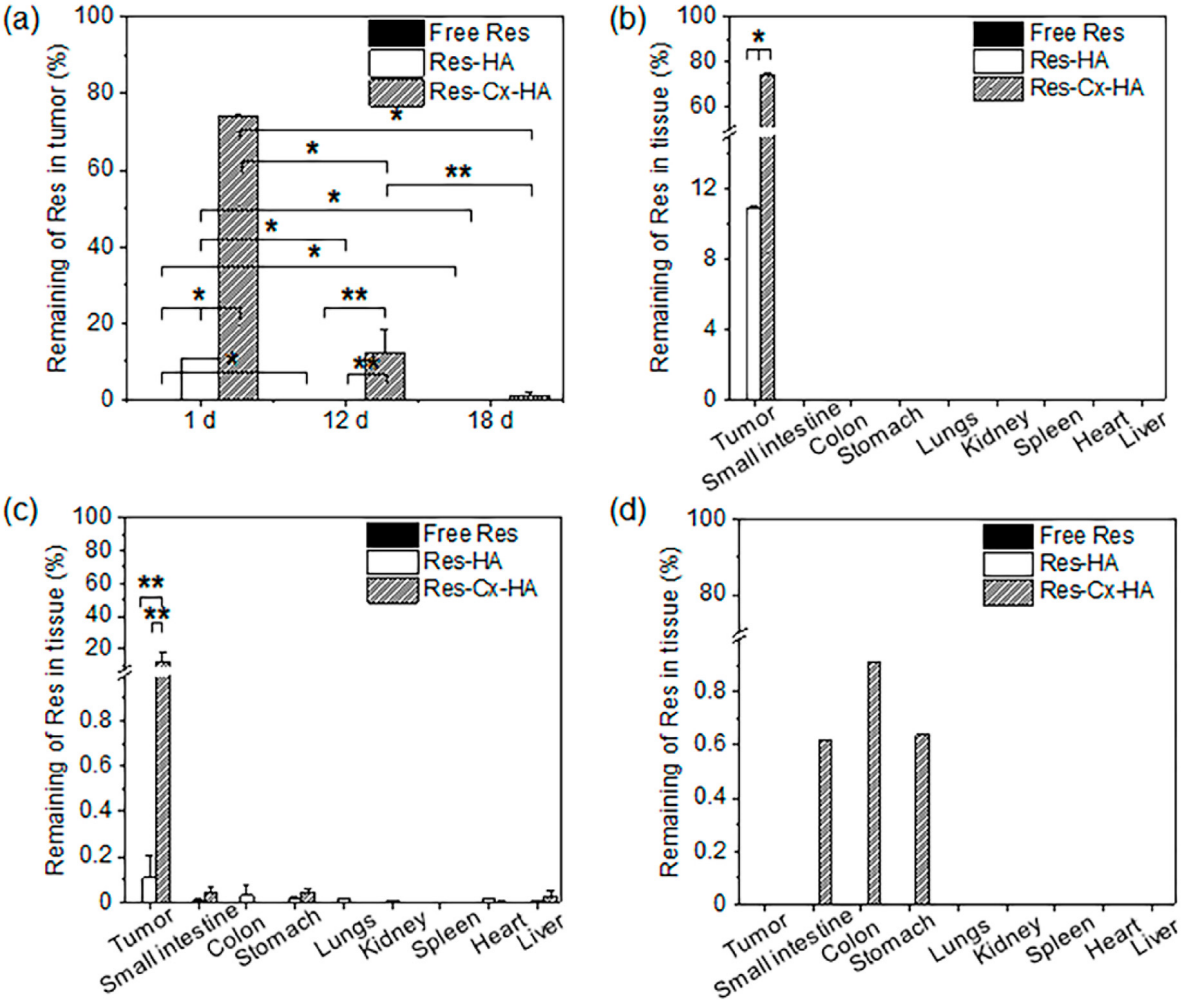
(a) Distribution of Res in tumors after intratumoral injection of Res, Res-HA, and Res-Cx-HA on days 1, 12, and 18. Distribution of Res in tumors and organs after intratumoral injection of Res, Res-HA, and Res-Cx-HA on days (b) 1, (c) 12, and (d) 18 (∗∗*p* ​< ​0.05, ∗*p* ​< ​0.001).

To examine the biodistribution of Res, the tumors and various organs were collected 1, 12, and 18 days after injection of the control, Res, Res-HA, and Res-Cx-HA solutions. The amounts of Res in the tumors and the small intestine, colon, stomach, lung, kidney, spleen, heart, and liver were also quantified (Fig. 7).

After an injection of Res, only 0.5% of the Res was found in the tumor on day 1, indicating that almost all Res was lost after only one day. For the Res-HA injections, the amount of Res remaining in the tumor was 11% on day 1 and ​< ​1% on day 12. This indicates that only a small amount of Res could be maintained in the tumor. In contrast, after injection of Res-Cx-HA, 74% of Res was observed in the tumor on day 1, which was far higher than the retention rates of the Res and Res-HA groups (∗*p* ​< ​0.001). Furthermore, 12% of Res remained in the tumors on day 12; however, this proportion decreased to 1% on day 18 (∗*p* ​< ​0.001). This was likely due to the sustained Res release from the Res-Cx-HA, which was kept in the tumor for an extended period by the Cx-HA depot. Additionally, very small amounts of Res (<1% total injected Res) were found in the small intestine, colon, stomach, lung, kidney, spleen, heart, and liver in all Res treatments, including Res, Res-HA, and Res-Cx-HA.

Collectively, our results confirmed that direct intratumoral injection of Res-Cx-HA significantly enhances the distribution of Res in the tumor.

### 3.7 Histology studies

**Fig. 8:**
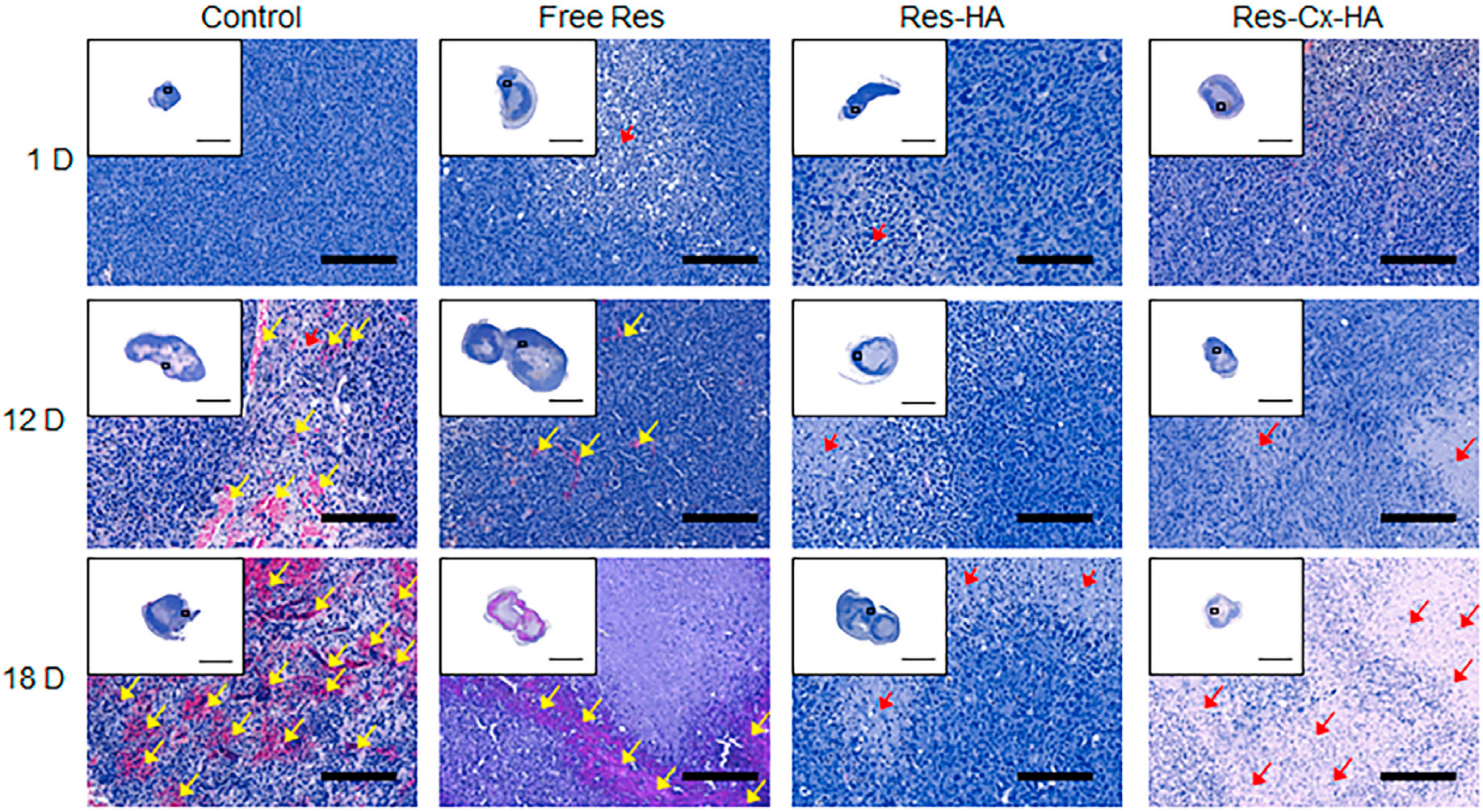
H&E staining of tumor sections on days 1, 12, and 18 after intratumoral injection of xenograft-bearing mice with Free Res, Res-HA, and Res-Cx-HA (Scale bar for the staining image in a white square box: 5000 ​μm; scale bar in enlarged images: 200 ​μm, Yellow and red arrows indicated blood vessels and necrosis respectively).

Histological sections of the tumors injected with control, Res, Res-HA, and Res-Cx-HA on days 1, 12, and 18 were examined after staining with H&E (Fig. 8). Few signs of necrosis were observed in the control. Furthermore, the number of blood vessels increased as the implantation time increased. Tumors injected with free Res exhibited a few regions with some degree of necrotic indices on day 1 compared to the control. This implies that free Res exhibits some antitumor activity. However, blood vessels were observed on day 12 in tumors injected with free Res because Res did not remain in the tumor. Tumors injected with Res-HA exhibited necrosis from day 1–12. However, small blood vessels were observed on day 12 in the tumors.

In contrast, Res-Cx-HA exhibited few blood vessels and higher necrosis rates on days 12 and 18. More importantly, the necrotic regions that were interspersed between viable tumor areas increased as implantation time increased. These results indicate that Res-Cx-HA persisted at the injected site for the full experimental period of 18 days.

**Fig. 9:**
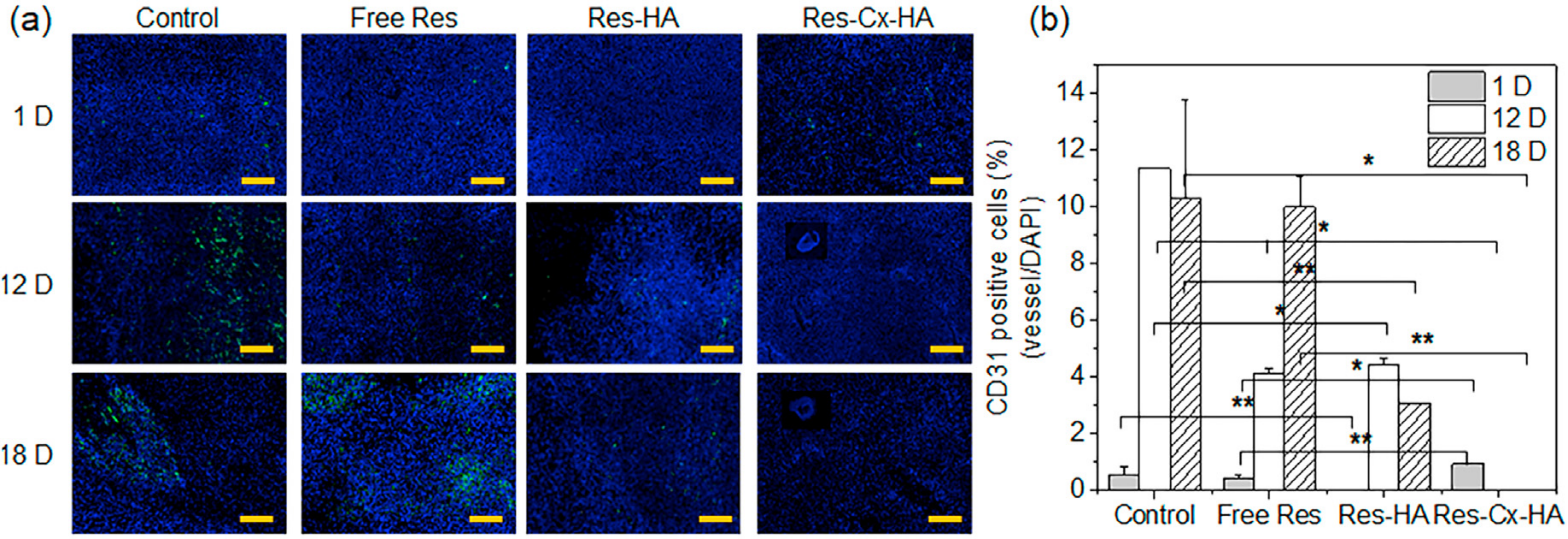
(a) Merged images of 4′,6-diamidino-2-phenylindole (DAPI; blue, nuclei) and CD31 (green, blood vessels cells) staining (scale bar: 200 ​μm) and (b) CD31-positive cells per DAPI in tumors on days 1, 12, and 18 after intratumoral injection of xenograft-bearing mice with Res, Res-HA, and Res-Cx-HA (∗∗*p* ​< ​0.05, ∗*p* ​< ​0.001).

The degree of angiogenesis in the tumors injected with saline (control), Res, Res-HA, and Res-Cx-HA was confirmed through merged images of CD31 green fluorescence (vascular) and blue DAPI fluorescence (nuclei) (Fig. 9; full staining tumor images are shown in Figure S5).

After 1, 12, and 18 days, the control exhibited clear blue fluorescence corresponding to the nucleus DNA stain in the tumor, and green fluorescence corresponding to vascular cells did not differ significantly from other groups on day 1 but increased sharply on day 12 compared to other groups. For tumors injected with Res and Res-HA, green fluorescence gradually increased over time. In contrast, the green fluorescence of tumors injected with Res-Cx-HA gradually decreased with time. The merging of blue and green fluorescence indicates regions with angiogenesis in the injected control, Res, Res-HA, and Res-Cx-HA groups. A small amount of green fluorescence was seen in the tumors injected with Res-Cx-HA, indicating that angiogenesis was strongly inhibited.

CD31-positive cells were counted and normalized to the total stained tissue area to determine the degree of angiogenesis. The control group exhibited rapid and high angiogenesis, reaching rates of 5%, 100%, and 90% on days 1, 12, and 18, respectively. In the group injected with Res only, blood vessels (green, CD31-positive cells) accounted for 3% of the total stained area on day 1 and increased to 36% and 88% with transplantation time. The group treated with Res-HA showed the lowest degree of angiogenesis (0.4%) on day 1, and increased to 39% and 27% on days 12 and 18, respectively. In the case of intratumoral injection of Res-Cx-HA, angiogenesis was continuously suppressed by 8% on day 1, reaching levels below 0.1% on days 12 and 18. Collectively, our findings indicated that prolonged sustained release of Res from Cx-HA may reduce angiogenesis inside the tumor.

**Fig. 10:**
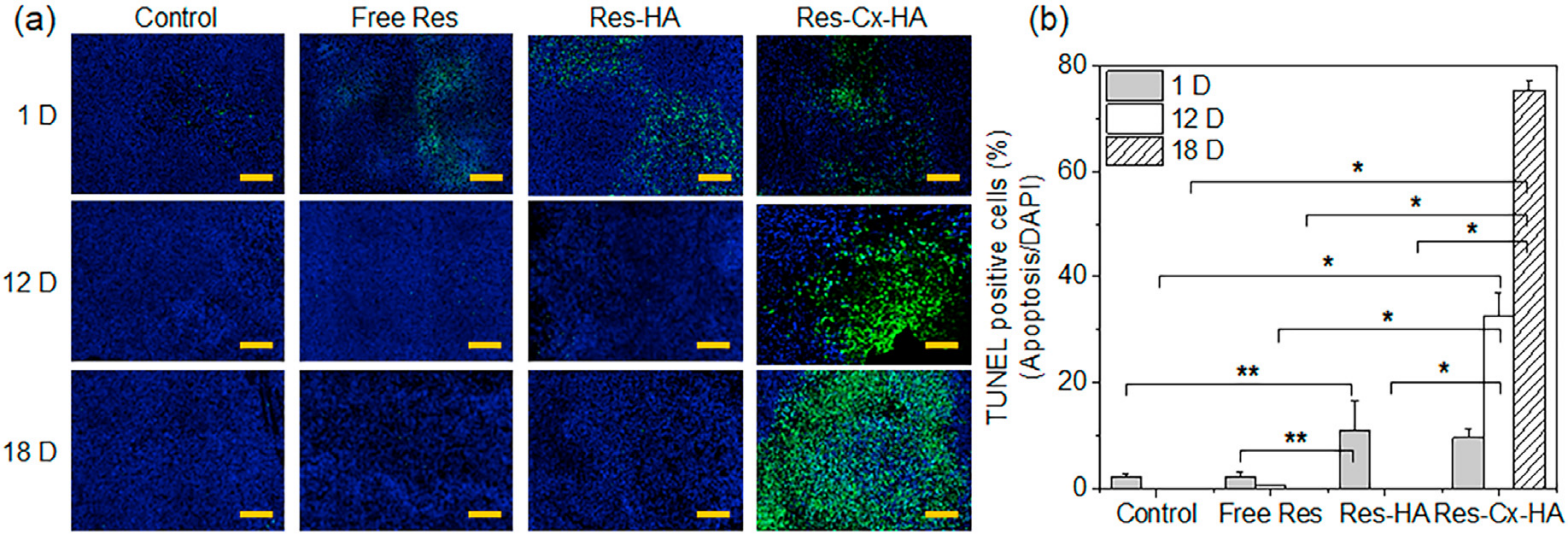
(a) Merged images of 4′,6-diamidino-2-phenylindole (DAPI; blue, nuclei) and TUNEL (green, apoptotic cells) staining (scale bar: 200 ​μm) and (b) TUNEL positive cells per DAPI in tumors on days 1, 12, and 18 after intratumoral injection of xenograft-bearing mice with Res, Res-HA, and Res-Cx-HA (∗∗*p* ​< ​0.05, ∗*p* ​< ​0.001).

The blue DAPI fluorescence (nuclei), green TUNEL fluorescence (apoptosis), and a merger of the images for tumors treated with control, Res, Res-HA, and Res-Cx-HA after 1, 12, and 18 days are illustrated in Fig. 10a (full staining tumor-images shown in Figure S6). The TUNEL-positive cells were counted and normalized to the total stained tissue area to determine the extent of apoptosis (Fig. 10b).

In control tumors, bright blue fluorescence corresponding to the nuclei of live cells was evident, whereas green fluorescence from TUNEL staining was undetectable. The percentage of apoptotic cells (TUNEL-positive cells) in the control group was below 3% on day 1 and no positive cells were detected on days 12 and 18.

For the tumors injected with Res and Res-HA, green fluorescence was observed on day 1 but was no longer detected on day 12. The percentages of apoptotic cells in the groups injected with Res and Res-HA were 3% and 14% on day 1, respectively, and decreased to <1% with implantation time.

Furthermore, the Res-Cx-HA group exhibited green TUNEL fluorescence throughout the entire experimental period and green fluorescence increased as the implantation time increased. The percentage of apoptotic cells on day 1 was 13% and rapidly increased to 43% on day 12 and 99% on day 18, which was attributed to the sustained release of Res from Res-Cx-HA. This finding indicated that the sustained release of Res from Res-Cx-HA promoted the apoptosis of the tumor cells for 18 days.

**Fig. 11:**
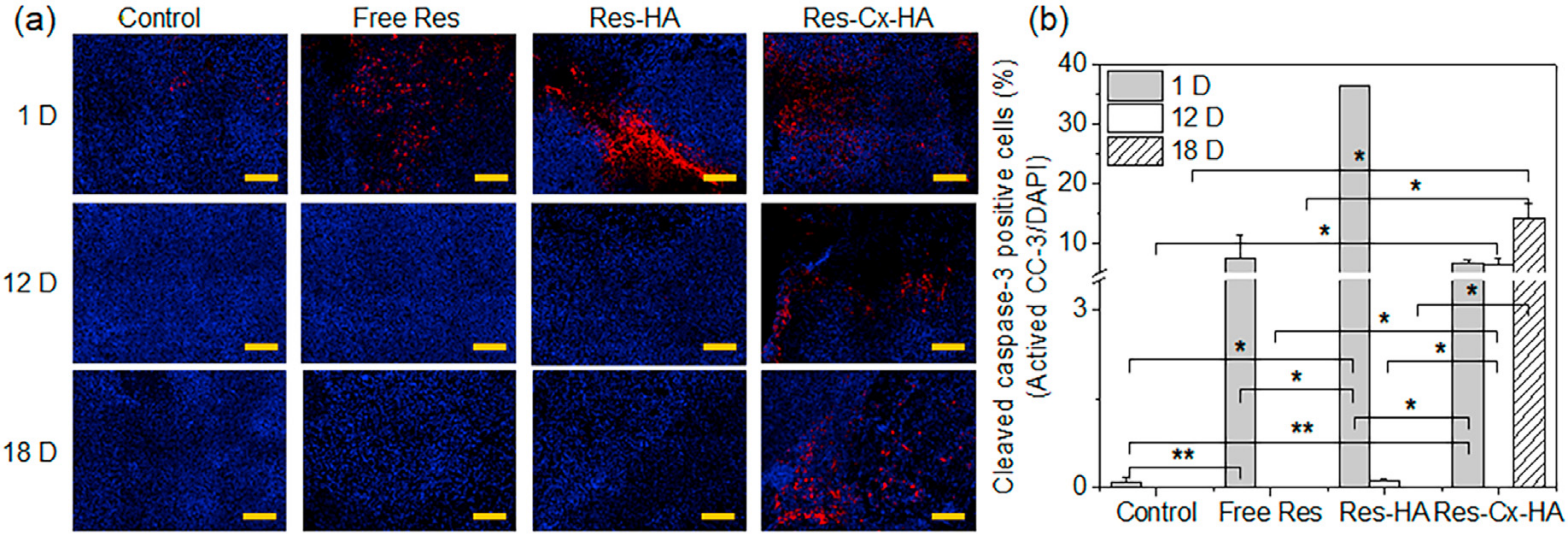
(a) Merged images of 4′,6-diamidino-2-phenylindole (DAPI; blue, nuclei) and cleaved caspase-3 (CCP-3, red) staining (scale bar: 200 ​μm) and (b) CCP-3-positive cells per DAPI in tumors on days 1, 12, and 18 after intratumoral injection of xenograft-bearing mice with Res, Res-HA, and Res-Cx-HA (∗∗*p* ​< ​0.05, ∗*p* ​< ​0.001).

For tumors injected with control, Res, Res-HA, and Res-Cx-HA, blue DAPI fluorescence (nuclei), red fluorescence from cleaved caspase-3 (CCP-3)-positive cells (apoptosis), and the combined images of DAPI and CCP-3 are presented in Fig. 11 (full staining tumor images are shown in Figure S7). The degree of apoptosis was confirmed by staining of CCP-3-positive cells.

After 1, 12, and 18 days, the control showed clear blue fluorescence corresponding to the nucleus DNA stain in the tumor, and almost no red fluorescence corresponding to CCP-3. For tumors injected with Res and Res-HA, red fluorescence was evident as early as day 1 but decreased with time. In contrast, the red fluorescence of the tumors injected with Res-Cx-HA persisted on days 1 and 12 and then doubled on day 18. The merged blue and red fluorescence images represented an area showing CCP-3-positive cells in the injected control, Res, Res-HA, and Res-Cx-HA groups. Particularly, red fluorescence increased over time in Res-Cx-HA-injected tumors, suggesting that there is a clear and distinct effect of apoptosis through CCP-3, which is activated last in the apoptosis process.

The CCP-3-positive cells were counted and normalized to the total stained tissue area to determine the degree of CCP-3-positive cells. In the group injected with free Res, CCP-3-positive cells with red fluorescence accounted for 20% of the stained tissue area on day 1 and decreased to less than 0.1% with increasing transplantation time. The apoptotic cells of the group treated with Res-HA showed the highest CCP-3 activity, reaching 100% on day 1, but then decreased with transplantation time. In the case of intratumoral injection of Res-Cx-HA, the red fluorescence of CCP-3-positive cells was 18% on day 1, 17% on day 12, and 39% on day 18, thus confirming that CCP-3 remained active throughout the entire experimental period. Collectively, our findings indicated that sustained release of Res from Cx-HA may significantly increase the CCP-3 activity of tumor cells, thereby promoting long-term apoptosis.

## 4 Discussion

Res is well known as a naturally occurring polyphenol with several therapeutic properties such as cardioprotective, neuroprotective, chemopreventive, and antiaging activities. Several *in vitro* and *in vivo* studies have assessed the effectiveness of Res as a candidate treatment against breast cancer, lung cancer, prostate cancer, pancreatic cancer, and leukemia [7,8].

However, one of the challenges of using Res in clinical cancer therapy is to achieve adequate bioavailability at a tolerable dose, as this compound exhibits a short half-life, chemical instability, and low bioavailability when administered in various ways. In previous work, at 6 ​h after intragastric administration of Res in pig, most of Res metabolized to Res derived metabolites and approximately only 15% of the initial Res administered was recovered in the pig intestine [37]. Additionally, Res in rats after single-dose tail vein injection administration of Res excreted almost over 80% in the urine within 4 ​h [38].

Among several delivery systems to overcome the poor bioavailability and poor pharmacokinetics of Res, our study explored intratumoral delivery, as this approach could provide an ideal drug delivery platform compared to other conventional routes of administration such as continuous intravenous infusion or high-dose infusions.

Several types of hydrogels have been widely utilized for the intratumoral delivery of Res [32,39,40]. Several groups have reported the good biocompatibility of crosslinked HA using carbodiimide series, (metha)acrylate series, diglycidyl ether, etc [[41], [42], [43]]. Among them, click-crosslinkable hydrogels remain in liquid form prior to injection but solidify through click-crosslinking during injection, allowing for the generation of a minimally invasive drug depot.

Meanwhile, we found that Cx-HA obtained from click-crosslinking between HA-TCO and HA-Tet has good biocompatibility for common tissues [34,35,44] (Figure S8). Therefore, Res-HA-TCO and Res-HA-Tet formulations in this study were easily prepared. The key requirements for intratumoral delivery and more specifically for *in-situ* depot-forming of click-crosslinkable formulations for Res delivery were the following: (1) injectability into the tumor tissue and (2) formation of a depot inside the tumor that could remain at the injected site for extended periods.

The injectability of HA-Tet and HA-TCO formulations with and without Res through standard gauge needles using dual-barrel syringes was verified by evaluating the evenness of injection flow and a lack of clogging of the syringe needle until all of the HA-Tet and HA-TCO loaded in the dual-barrel syringe were depleted. Depot formation inside the tumor was rapidly achieved after intratumoral injection using the dual-barrel syringe. The formed Cx-HA depot revealed an interconnected porous network structure that allowed for Res diffusion. The click-crosslinking-formed depot must also be maintained inside the tumor for prolonged periods to ensure sustained drug delivery. In this study, the Cx-HA depot inside the *in vivo* tumor persisted for at least 18 days, as demonstrated by NIR monitoring, whereas HA alone did decrease with implantation time. Although we did not examine the identification of *in vivo* biodegraded materials, generally, HA can be degraded by a family of enzymes called hyaluronidases [45].

Importantly, the injectable Res-Cx-HA formulations examined herein were easily prepared, easily injected, showed good injectability into the tumor tissue, and Cx-HA successfully acted as a depot for Res inside the tumor and retained good structural integrity *in vivo*. Collectively, these findings suggest that the formulations tested herein are well suited for clinical use as promising candidates for the generation of Res depots.

Very few studies have examined injectable and cross-linkable formulations for intratumoral injection of Res depots. Therefore, our study sought to overcome the limitations of Res administration via continuous intravenous infusion or high-dose infusions by instead injecting Res-Cx-HA formulations directly into tumors. In this way, the bioavailability and therapeutic efficacy of Res can be improved by extending Res release from the Cx-HA depot within the tumor over a longer period.

In the present study, the Res-Cx-HA formulations were successfully injected into the centers of tumors using a 23-gauge needle to produce Res depots within the tumor. The availability of Res inside Cx-HA at the tumor site was maintained for extended periods, whereas Res was rapidly lost when paired with HA. Therefore, our findings confirmed that the injectable Cx-HA formulations designed in this study act as Res depots that allow for the sustained release of Res inside the tumor.

In this work, *in vitro* Res release from Res-HA was below 10% even on day 1 and the amount of Res remaining in the tumor after intratumoral injection of Res was below 1% even on day 1. This result was consistent with previous reports that Res has a short half-life (8–14 ​min) under physiological conditions [10,37,38].

Therefore, we concluded that the low Res amount in the tumor was due to the rapid metabolic degradation and/or rapid systemic elimination of Res from the tumor. In contrast, tumors injected with Res-HA contained 10% Res on day 1, meaning that this formulation increased the stability of Res in the tumor. Furthermore, tumors injected with Res-Cx-HA contained approximately 75% of Res on day 1. Importantly, Res inside Cx-HA was observed in tumors for at least 18 days and little or no Res was detected in other organs such as the intestine, colon, stomach, lung, kidney, spleen, heart, and liver. Taken together, our findings indicated that the Cx-HA depot maintained therapeutic Res concentrations and thus caused significant antitumor activity by providing sufficient Res exposure inside the tumor compared to free Res and Res-HA.

Res exerts antiangiogenic properties, which inhibits the process of cancerous invasion into deeper tissues. H&E histology demonstrated that the tumors injected with Res-Cx-HA exhibited clear necrosis and almost no blood vessels. Additionally, the Res-Cx-HA depot reduced CD31 expression compared with the Res and Res-HA treatments. These observations indicate that angiogenesis was almost completely suppressed by the long-lasting release of Res from the Cx-HA depot.

Moreover, TUNEL and CCP-3 assays revealed large areas of apoptotic cells in tumors injected with Res-Cx-HA. In contrast, little to no apoptotic cells were observed during the experimental period in tumors injected with Res or Res-HA. Based on this distribution pattern of apoptotic cells, we concluded that intratumoral injection of Res-Cx-HA would cause significant antitumor activity via sufficient exposure of Res inside the tumor compared to free Res and Res-HA treatment.

Meanwhile, the primary clinical treatment for TNBC is surgical removal and then followed by systemic chemotherapy or local irradiation therapy to completely prohibit recurrence of TNBC. Many anticancer drugs including Res have been used in clinical chemotherapy. Therefore, Res-Cx-HA could be intratumorally injected in surgical removal cavity of the TNBC to completely eliminate residual TNBC and/or prevent the recurrence of TNBC.

## 5 Conclusion

Our findings demonstrated that Res-Cx-HA depots successfully formed in the tumor after intratumoral injection and maintained Res release for an extended period. Based on the results of the intratumoral injection experiments, we concluded that Res-Cx-HA could induce long-lasting Res release and significantly inhibit tumor growth *in vivo*. Therefore, intratumoral injection of Res-Cx-HA constitutes a promising treatment for TNBC patients. A more detailed release kinetics of Res from Res-Cx-HA and *in vivo* anti-tumor efficacy of Cx-HA and Res-Cx-HA using large animal are planned as future work.

## Author contribution

Conceptualization, G.R.S., H.E.K., and M.S.K.; Formal analysis, G.R.S., H.E.K., and H.J.J.; Formal analysis, G.R.S., H.E.K., H.J.J., J.H.K., H.S.C., and M.S.K.; Methodology, G.R.S., H.E.K., H.J.J., J.H.K., S.C., and M.S.K.; Validation, G.R.S., H.E.K., H.J.J., H.S.C., and M.S.K.; Visualization, G.R.S., H.E.K., and H.J.J.; Funding acquisition, S.C., H.S.C., and M.S.K.; Project administration, S.C., H.S.C., and M.S.K.; Supervision, M.S.K.; writing—original draft, G.R.S.; writing—review and editing, M.S.K. All authors have read and agreed to the published version of the manuscript.

## Funding

This study was supported by the 10.13039/501100003725National Research Foundation of Korea (NRF) grants, Creative Materials Discovery Program (2019M3D1A1078938) and Priority Research Centers Program (2019R1A6A1A11051471).

## Ethical statement

We confirm that any aspect of the work covered in this manuscript that has involved experimental animals has been conducted with the ethical approval of all relevant bodies and that such approvals are acknowledged within the manuscript.

## Declaration of competing interest

The authors declare no conflict of interest.
