# Supplementary material for: Injectable click-crosslinked hydrogel containing resveratrol to improve the therapeutic effect in triple negative breast cancer
Source: Mater Today Bio. 2022 Aug 5;16:100386. doi: 10.1016/j.mtbio.2022.100386 (PMC9386493; doi:10.1016/j.mtbio.2022.100386)
Supplement: Multimedia component 1 [file mmc1.docx]

**Supporting Information**

**Injectable click-crosslinked hydrogel containing resveratrol to improve the therapeutic effect in triple negative breast cancer**

Gi Ru Shin, Hee Eun Kim, Hyeon Jin Ju, Jae Ho Kim, Sangdun Choi, Hak Soo Choi, Moon Suk Kim*

**Figure S1.** Schematic images for preparation of HA-TCO and HA-Tet using HA, TCO and Tet, and the Cx-HA formed by click-crosslinking between HA-TCO and HA-Tet.


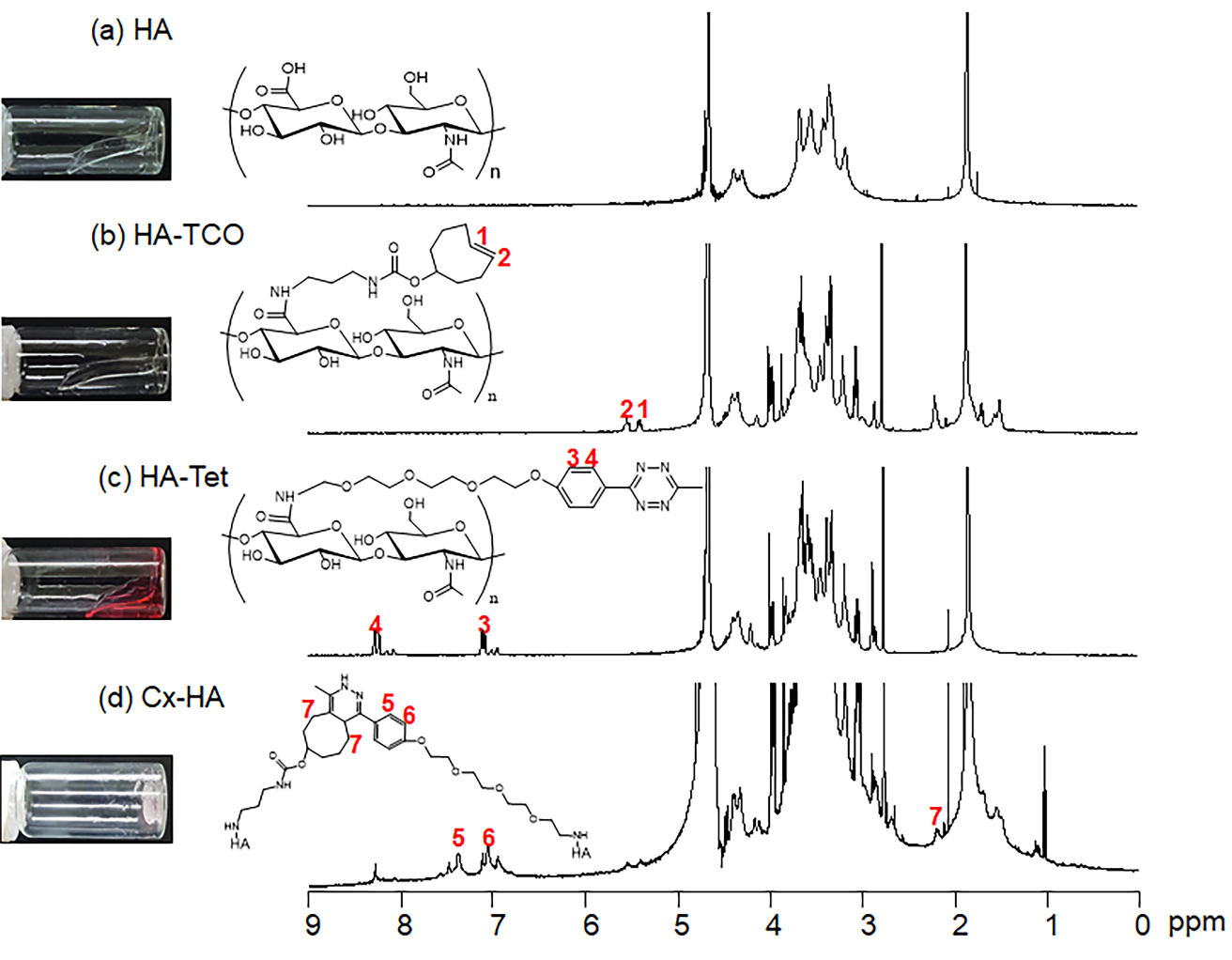


**Figure S2.** ^1^H-NMR spectra of (a) HA, (b) HA-TCO, (c) HA-Tet and (d) Cx-HA.

*
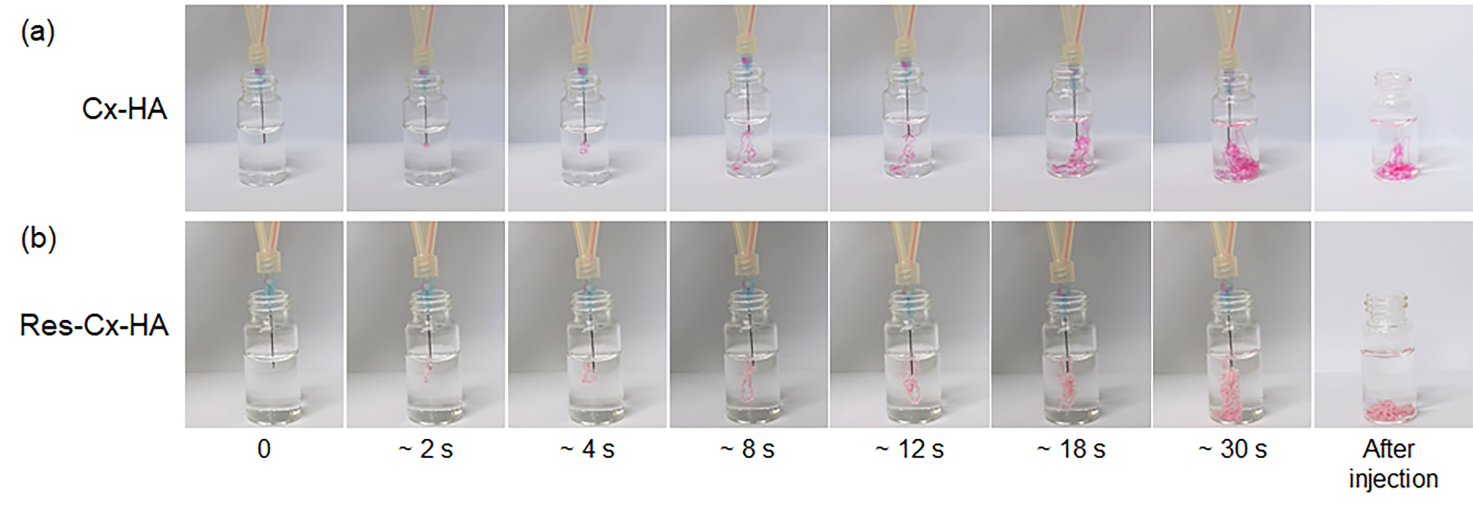
*

**Figure S3**. Photo images for pushing of HA-Tet and HA-TCO with and without Res through a 23 G syringe needle from each compartment of a dual-barrel syringe without clogging in PBS for 30 s.


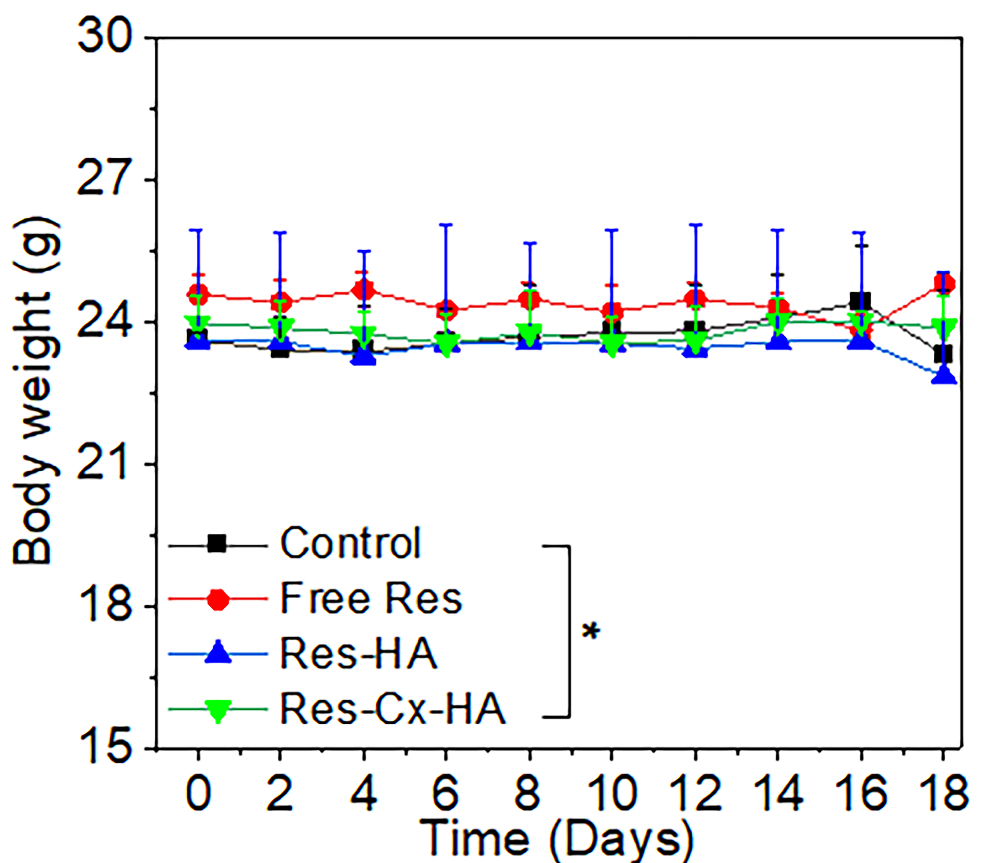


**Figure S4.** Body weight of control, Free Res, Res-HA and Res-Cx-HA (* *p* > 0.05 for Free Res, Res-HA and Res-Cx-HA versus the control at each day for 18 days).


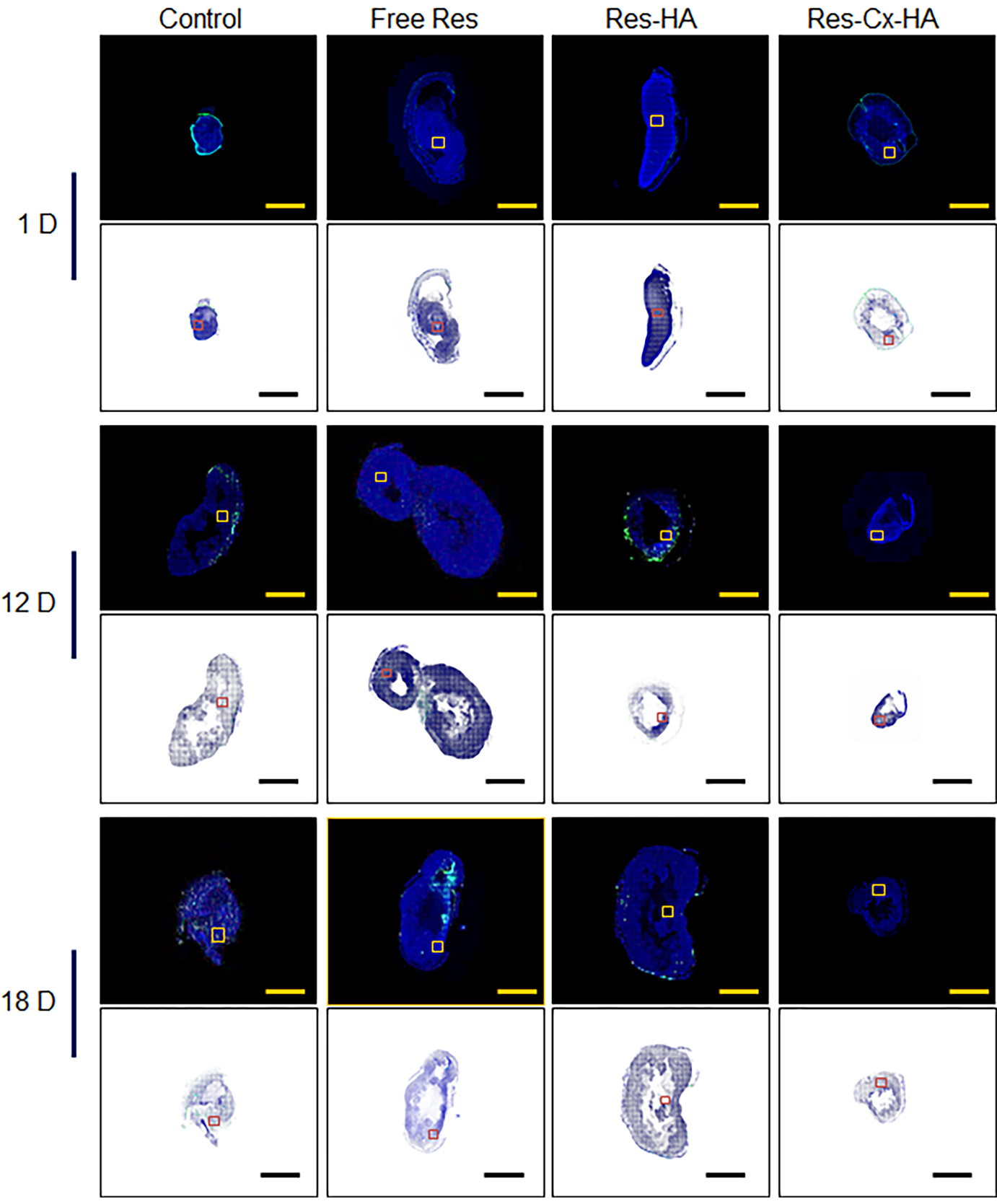


**Figure S5**. Full tumor images. Merged images of 4',6-diamidino-2-phenylindole (DAPI; blue, nuclei) and CD31 (green, blood vessels cells) staining (scale bar for the staining image in the black and white backgrounds: 5000 μm) in tumors on days 1, 12, and 18 after intratumoral injection of xenograft-bearing mice with Res, Res-HA, and Res-Cx-HA (the staining image of Res at 12 days is presented in two pieces because it was pushed aside during production of the staining block).


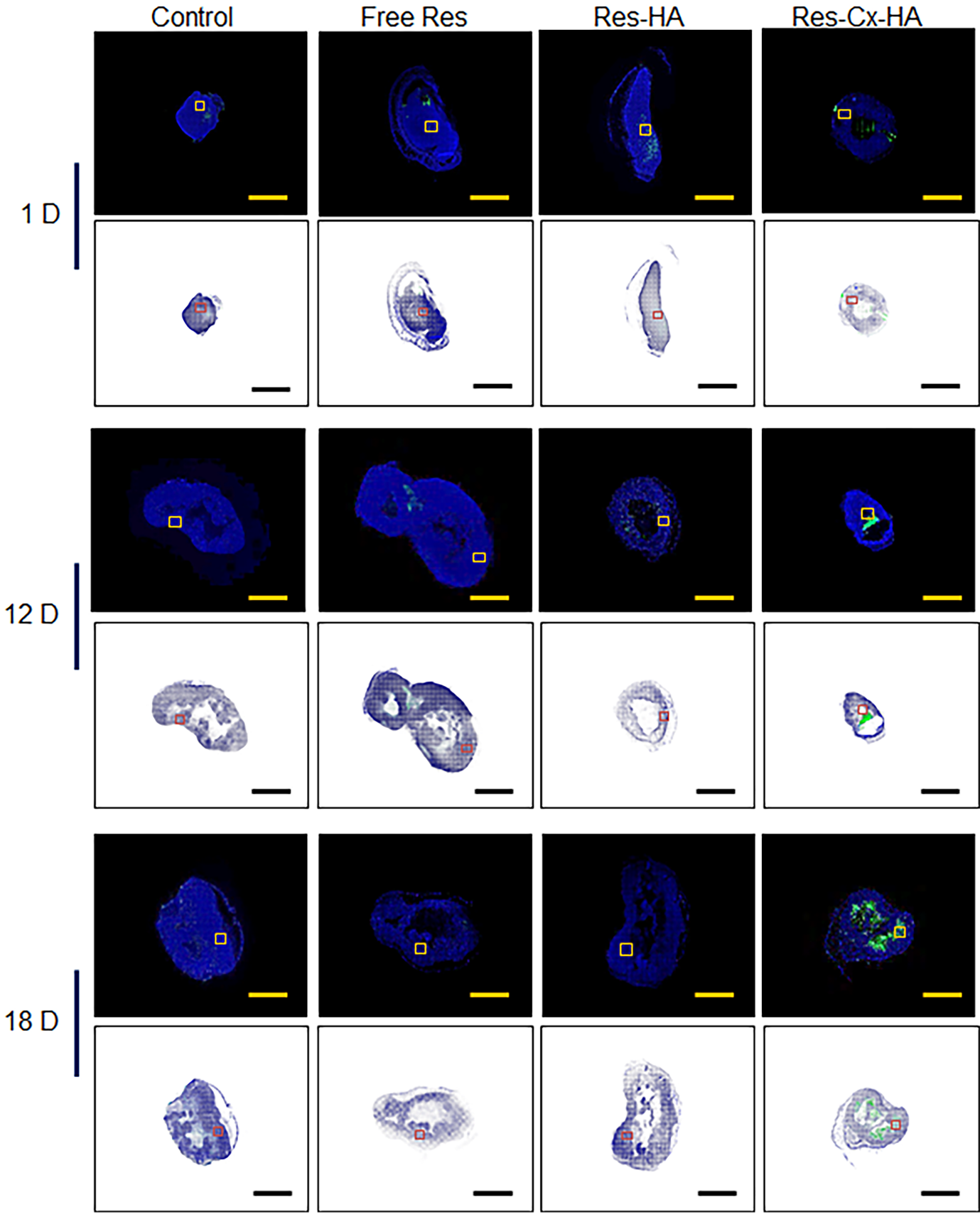


**Figure S6**. Full tumor images. Merged images of 4',6-Diamidino-2-phenylindole (DAPI) staining (blue, nuclei) and TUNEL (green, apoptotic cells) staining (scale bar for the staining image in the black and white backgrounds: 5000 μm) in tumors on days 1, 12, and 18 days after intratumoral injection of xenograft-bearing mice with an injection of free Res, Res-HA and Res-Cx-HA (the staining image of free Res at 12 days is presented in two pieces because it was pushed aside during production of the staining block).


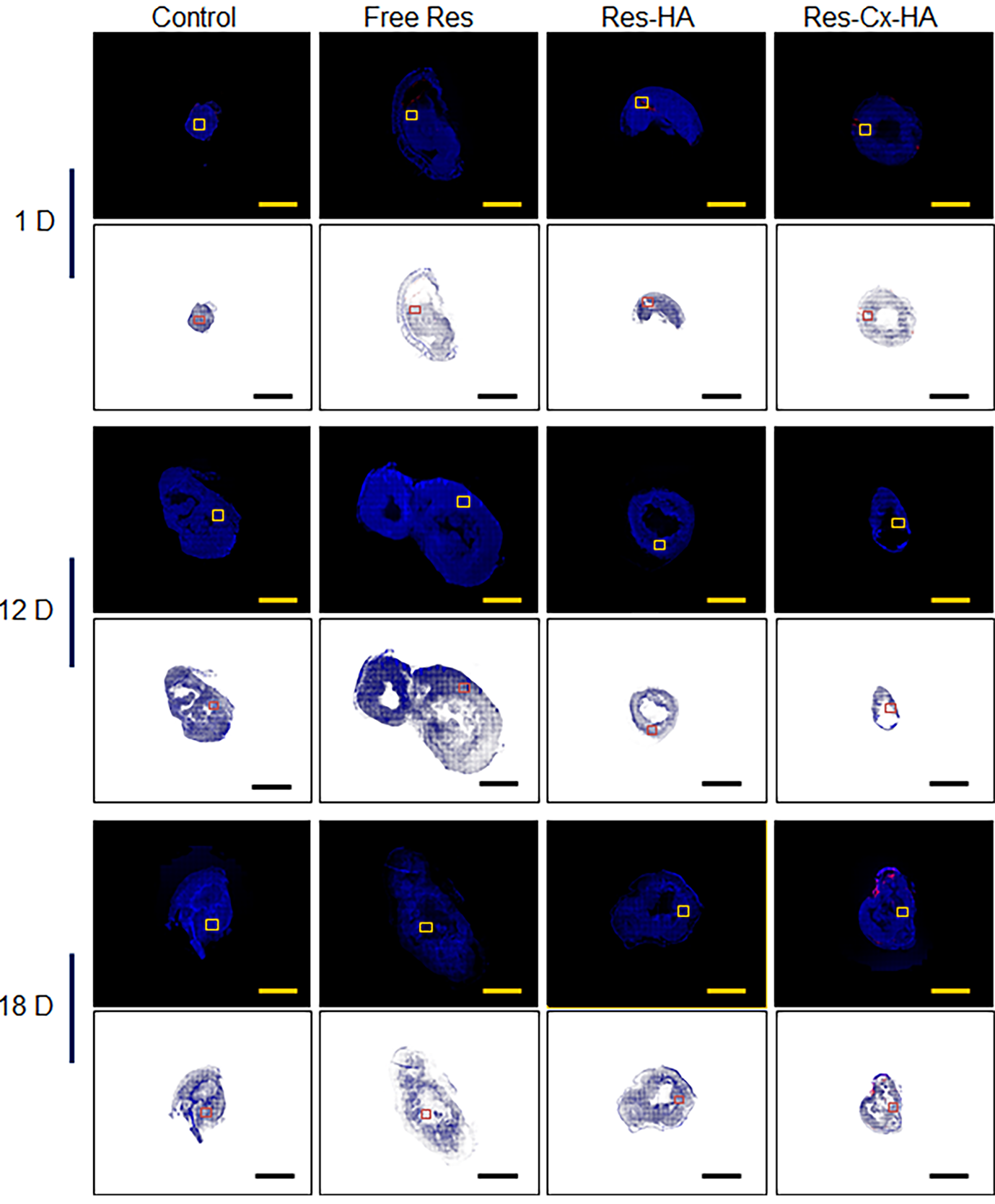


**Figure S7**. Full tumor images. Merged images of 4',6-diamidino-2-phenylindole (DAPI) staining (blue, nuclei) and cleaved caspase-3 (CCP-3, red) staining (scale bar for the staining image in the black and white backgrounds: 5000 μm) in tumors on days 1, 12, and 18 days after intratumoral injection of xenograft-bearing mice with an injection of free Res, Res-HA and Res-Cx-HA (the staining image of free Res at 12 days is presented in two pieces because it was pushed aside during production of the staining block).


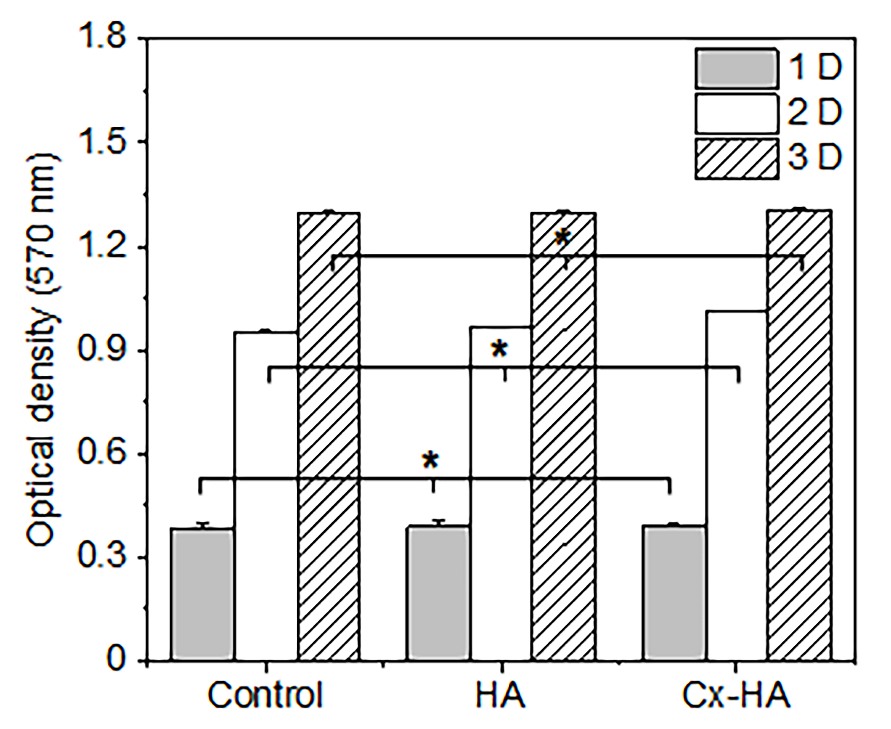


**Figure S8**. MTT assay results of *in vitro* viability of MDA-MB-231 cell line in well plate (control), HA and Cx-HA after 1, 2, and 3 days (**p* > 0.05). *In accordance with one referee’s suggestion, biocompatibility study of the Cx-HA hydrogels was performed. HA and Cx-HA hydrogel showed almost similar viability of MDA-MB-231 cell line in well plate for 3 days. This result implies that the Cx-HA hydrogels prepared in this work showed good biocompatibility.
